# Senescent Cell Heterogeneity: Tissue-Dependent Signatures and Age Dynamics Revealed by Human scRNA-Seq Data

**DOI:** 10.3390/ijms27188416

**Published:** 2026-09-21

**Authors:** Kseniia Matveeva, Daniil Shevyrev

**Affiliations:** 1 Research Center for Genetics and Life Sciences, Sirius University of Science and Technology, 354340 Sirius, Russia; 2 Research Center for Translational Medicine, Sirius University of Science and Technology, 354340 Sirius, Russia

**Keywords:** single-cell RNA sequencing, senescence, human senescent cell mapping, senescent cell heterogeneity, senescent cell age dynamic, SenePy, SenMayo, GSEA, DEG, senescence signature

## Abstract

It is well established that aging is accompanied by the accumulation of senescent cells across tissues. The triggers inducing cellular senescence are diverse and vary across cell types and organs, underpinning the high diversity of senescence phenotypes within individual cell types as well as at the tissue and organismal levels. Identifying common transcriptional patterns and senescence triggers while accounting for the remarkable diversity of aged and dysfunctional cells is fundamental to developing strategies aimed at reducing senescent cell burden. The aim of this study was to investigate the heterogeneity of human senescent cells across tissues at single-cell transcriptome resolution. We selected publicly available scRNA-seq data from healthy donors for the skin, lungs, small and large intestine, kidneys, and heart. The final dataset comprised 1.5 million cells from six tissues, encompassing 64 annotated cell types across 177 donors aged 15 to 88 years. To identify cell type-specific senescence signatures, we reproduced and adapted the signature identification algorithm underlying the SenePy library. This enabled us to identify de novo genes associated with aging and senescent status within a given dataset. Next, for senescent cells of each cell type, we obtained differentially expressed gene sets and performed gene set enrichment analysis (GSEA) using a broad collection of terms reflecting functional status, metabolic activity, and adaptive responses to various forms of cellular stress. As a result, we identified organ-specific aging features in senescent cells of different cell types. We also assessed the enrichment of antigen presentation pathways and the production of senescence-associated pro-inflammatory factors (SASP) in senescent cells across tissues. Furthermore, our analysis revealed novel tissue-specific patterns of ligand-receptor interactions between senescent cells and their microenvironment.

## 1. Introduction

Tissue aging is a complex and multifaceted process. Because repair mechanisms are not perfectly efficient, damage accumulates over time at multiple levels—from molecular to tissue and organ level—and ultimately produces the aging phenotype of the whole organism [1,2]. Genetic, environmental, and behavioral factors determine the balance between tissue functional reserve and stress resilience [3]. Cell type-specific functions and unique local exposures in different tissues shape distinct profiles of cellular vulnerability to damage, producing high heterogeneity in aging phenotypes. Although no universal, widely accepted classification of cellular senescence exists, senescence broadly falls into physiological, stress-induced, and secondary forms [4,5]. Physiological senescence occurs during tissue development or wound healing, remains transient, and supports morphogenesis while preventing fibrosis [6]. Stress-induced senescence develops in response to macromolecular damage, contributes to age-related tissue changes, and underlies pathologies of old age [7,8]. Secondary senescence develops in normal cells under the influence of SASP factors [9,10,11]. Chronic stress-induced senescence can arise from replicative exhaustion (RS), oncogene activation (OIS), metabolic dysfunction (MS), or other genotoxic factors (GTS) [12,13,14,15,16]. Although the boundaries between these categories are somewhat arbitrary, specific senescence triggers activate characteristic transcriptional patterns and gene cascades. For example, replicative exhaustion and associated telomere shortening trigger a DNA double-strand-break response, activating the p53/p21 pathway, inhibiting CDK2/cyclin E, and inducing G1/S cell-cycle arrest, a hallmark of RS [17,18,19,20]. Activation of oncogenes such as KRAS or BRAF, or loss of PTEN, excessively stimulates mitogenic pathways and, through transcription factors ETS2 or BLIMP1, activates the p16INK4a/pRb pathway, thereby nearly irreversibly arresting proliferation and driving cells into an OIS state [21,22,23,24]. Genotoxic agents that damage DNA activate the single- and double-strand-break sensors ATM and ATR, which phosphorylate the effector kinases CHK1 and CHK2, thereby activating the p53/p21 pathway and inducing cell-cycle arrest, a hallmark of GTS [25,26]. Metabolic disturbances, together with chronic energy and oxidative stress, dysregulate the AMPK and SIRT1 sensors and activate the p53/p21 and p38/MAPK pathways, also leading to proliferation arrest and establishing the MS senescence phenotype [27,28,29]. Notably, despite the diversity of triggers and routes into senescence, these processes converge on two evolutionarily conserved pathways: p53/p21 and p16/pRb. Activation of p53/p21 generally represents a rapid primary stress response and may be reversible in some cases. In contrast, p16/pRb activation marks chronic stress and irreversible senescence [30,31,32]. Regardless of the specific pathway, entry into senescence culminates in permanent cell-cycle arrest, resistance to apoptosis, and secretion of inflammatory SASP factors, which can then induce secondary senescence in neighboring cells within the microenvironment [10,15,16,19].

Researchers commonly link the development of age-related diseases to the accumulation of senescent cells [33,34]. Understanding senescent-cell heterogeneity, identifying common features of senescent cells, and deciphering the trajectories by which cells become senescent under physiological and pathological conditions are important for developing technologies that reduce senescent cell burden and promote healthy longevity. Single-cell RNA sequencing (scRNA-seq) offers broad opportunities for studying tissue aging and elucidating how senescent cells interact with healthy cells in tissues. Using publicly available scRNA-seq data from multiple tissues of apparently healthy donors of different ages, we aimed to investigate the heterogeneity of senescent cells across tissues, determine the rate at which senescent cells accumulate in different cell types, and identify common patterns of cellular aging. In addition, we examined local interactions between senescent and normal cells across tissues to identify possible pathways of senescence induction in the microenvironment.

**Figure 1:**
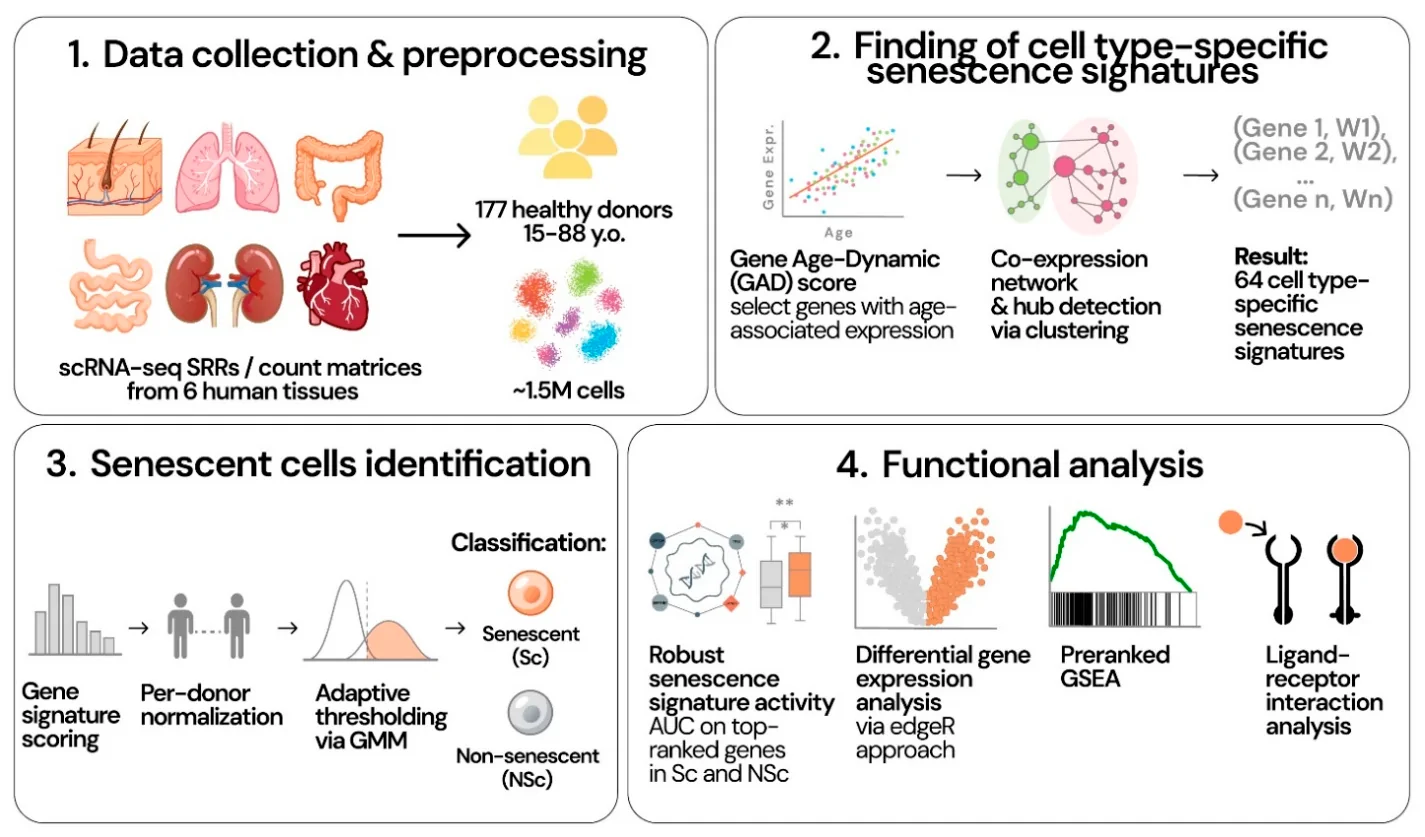
Schematic representation of the data processing and result generation pipeline employed in this study.

Notably, all downstream analyses were performed on the original, full-depth datasets to preserve data complexity, without any additional downsampling. The complete bioinformatics pipeline utilized in this study is schematically illustrated in Figure 1.

## 2. Results

### 2.1. Age-Related Dynamics of Senescent Cell Abundance Across Tissues

**Figure 2:**
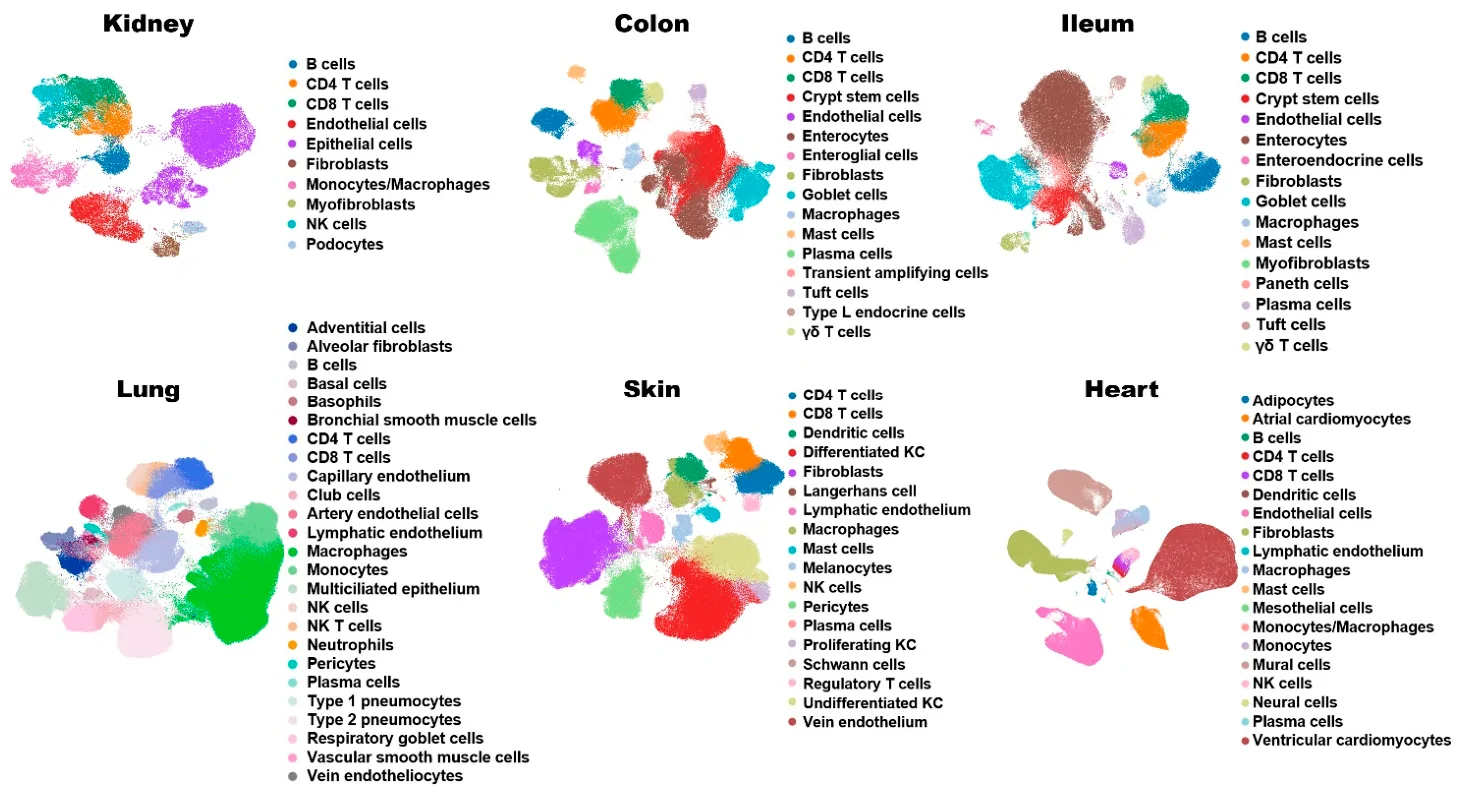
UMAP visualization of annotated cell types. Individual panels represent the cellular composition of the Kidney, Colon, Ileum, Lung, Skin, and Heart, color-coded by annotated cell type.

This study focused on cell types annotated during scRNA-seq analysis of the skin, lungs, large and small intestines, kidneys, and heart (Figure 2).

We then applied the SenePy algorithm de novo to these data (see Section 4), which allowed us to derive unique aging signatures for each cell type that passed quality filtering [35]. Based on these signatures, we computed a senescence score for every cell and constructed the corresponding distributions. Senescent cells within each cell type were then identified using adaptive GMM thresholds (see Section 4).

**Figure 3:**
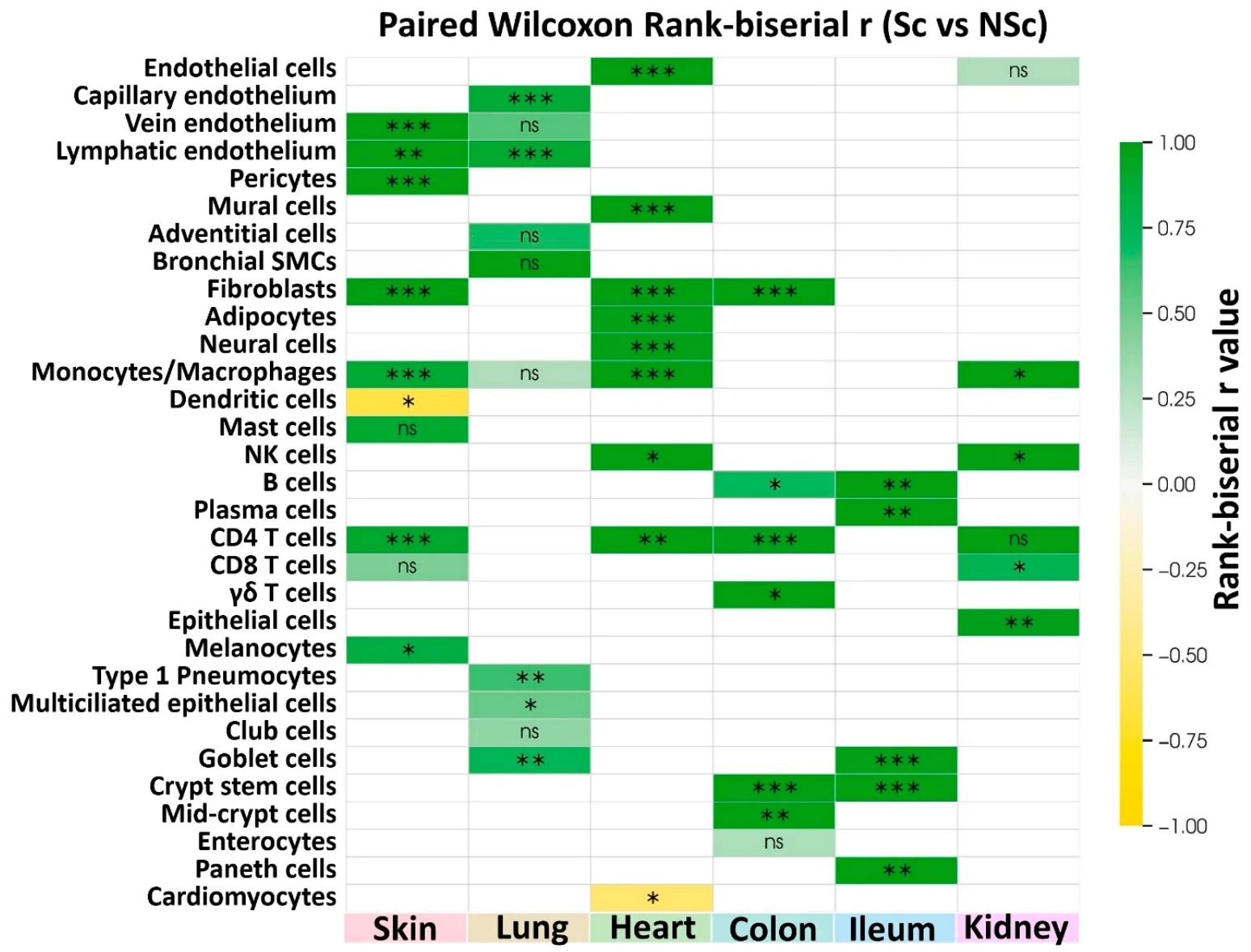
Validation of cellular senescence status via SenMayo signature enrichment analysis computed at single-cell resolution via AUCell. Heatmap displaying the effect size and statistical significance of the enrichment. Differences in AUCell enrichment scores between groups were quantified using the paired Wilcoxon rank-biserial correlation coefficient (r). *—*p* < 0.05, **—*p* < 0.01, ***—*p* < 0.001. ns—not significant statistically

The SenMayo gene set [36] is a widely recognized resource for defining transcriptional senescence signatures across tissues. Originally designed to detect hallmarks of aging across organ systems at the transcriptome level, it served as a natural benchmark in our analysis. We therefore assessed the enrichment of the SenMayo gene set in the senescent cells we identified relative to non-senescent cells of the corresponding cell types (Figure 3). Effect size analysis using the Wilcoxon rank-biserial correlation coefficient revealed significant enrichment of SenMayo genes in senescent cell populations for the vast majority of cell types across all tissues examined (Figure 3).

**Figure 4:**
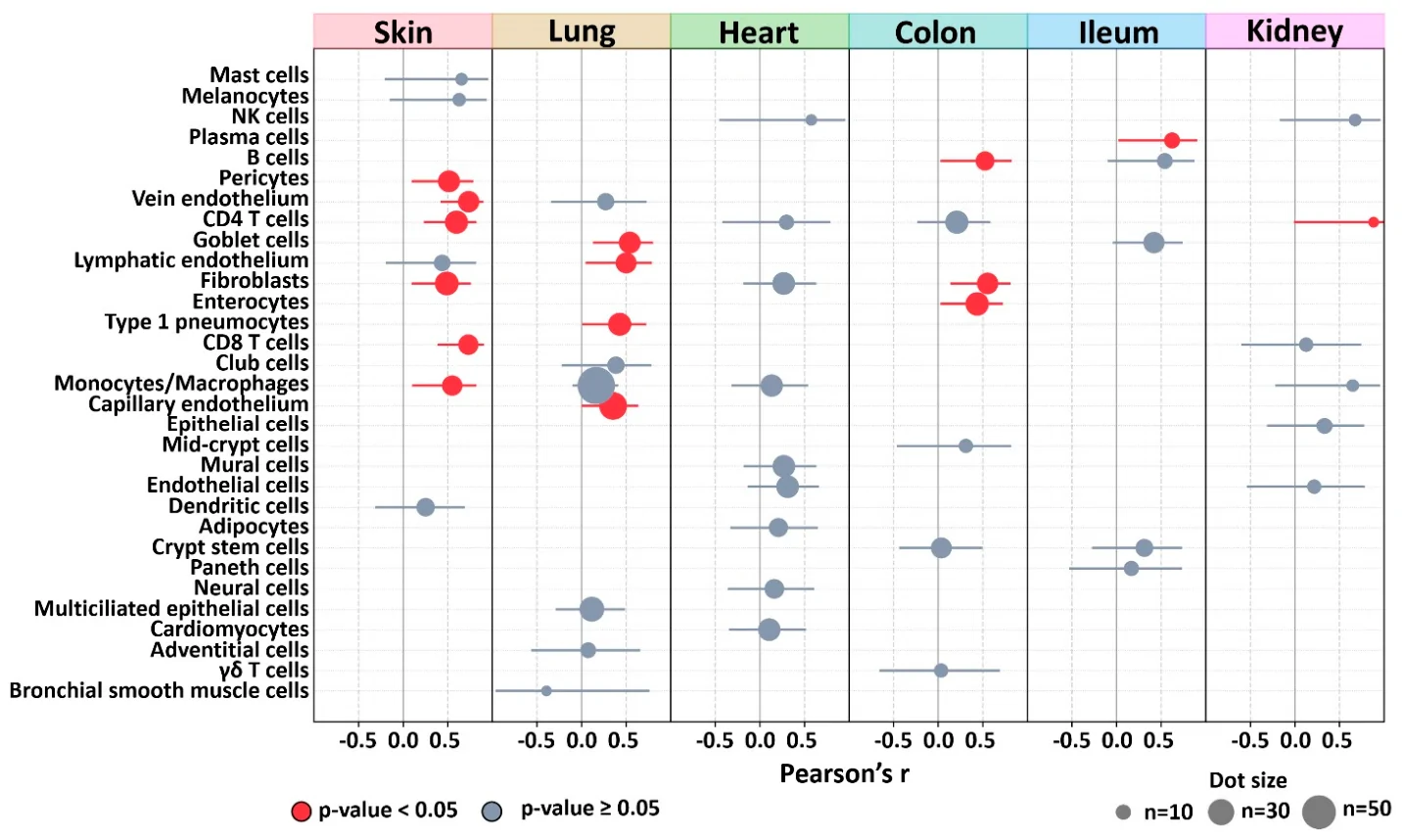
Correlation between age and the proportion of senescent cells across various cell types in different tissues. The forest plot displays Pearson correlation coefficients (r) along with their corresponding 95% confidence intervals for distinct cell types in the skin, lung, heart, colon, ileum, and kidney. Red dots and error bars highlights statistically significant correlations (*p* < 0.05). The size of each dot is proportional to the sample size (n).

To examine the heterogeneity and rate of aging across tissues, we analyzed correlations between donor age and the accumulation of senescent cells within annotated cell types (Figure 4). Notably, at this stage many cell types were excluded due to insufficient cell numbers. Our analysis revealed pronounced heterogeneity in age-dependent senescent cell accumulation across tissues. The strongest correlations were observed in the skin: age-related increases in the senescent cell fraction were most prominent in fibroblasts, venous endothelial cells, pericytes, monocyte-derived cells, and CD4^+^ and CD8^+^ T lymphocytes. In the lungs, a significant increase in senescent cell abundance was also detected among alveolar type I cells, capillary and lymphatic endothelial cells, and goblet cells. In the large intestine, the abundance of senescent fibroblasts, enterocytes, and B lymphocytes increased significantly with age, while senescent plasma cells accumulated in the ileum and senescent CD4^+^ T lymphocytes in the kidneys. Other cell types also exhibited a trend toward increasing senescent cell abundance with age, although these correlations did not reach statistical significance.

### 2.2. Transcriptional Heterogeneity of Senescent Cells Across Tissues

To investigate the activity of distinct transcriptional programs in senescent cells across tissues, we performed gene set enrichment analysis (GSEA) using gene sets associated with the activation of various biological programs. To minimize noise and mitigate the effects of scRNA-seq data sparsity, we aggregated single-cell profiles into pseudobulk samples for each cell type per donor. Reference gene sets were obtained from the MSigDB, Reactome, and GO Biological Process collections.

**Figure 5:**
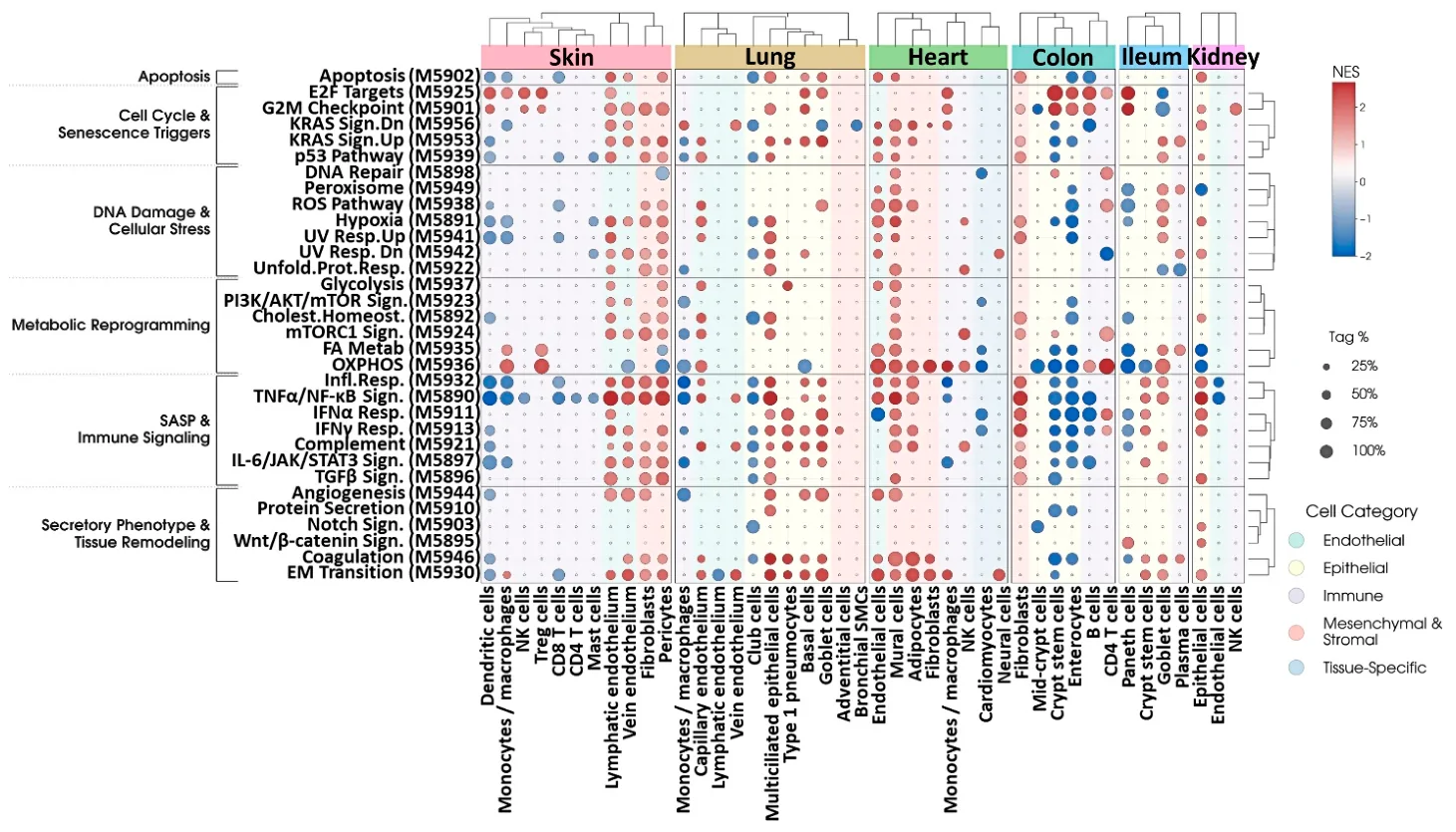
Gene set enrichment analysis (GSEA) of senescent versus non-senescent cells. Dot color indicates the normalized enrichment score (NES; red: enriched, blue: suppressed). Dot size represents the proportion of genes from each gene set forming the leading-edge subset that contributes to the enrichment signal. Pathways are grouped by functional categories.

From the MSigDB Hallmark collection, we selected gene sets reflecting the activity of apoptosis, cell cycle regulation, metabolic reprogramming, DNA damage response (DDR), immune signaling, and tissue remodeling programs (Figure 5). The analysis encompassed diverse cell types, including epithelial, endothelial, mesenchymal, immune, and tissue-specific specialized cells. This revealed tissue-specific enrichment patterns. In the skin, heart, and large intestine, cells of endothelial and mesenchymal origin showed the strongest enrichment of pathways related to cell cycle arrest (E2F Targets, G2/M Checkpoint, p53 Pathway), DDR and cellular stress (DNA Repair, ROS Pathway, Hypoxia), metabolic reprogramming (Glycolysis, PI3K/AKT/mTOR Signaling, mTORC1 Signaling), and SASP factor secretion. In contrast, in the lungs, small intestine, and kidneys, enrichment of SASP-associated inflammatory pathways was most pronounced and occurred predominantly in epithelial cells.

Additionally, certain cell types—endothelial and mesenchymal cells in the skin and heart, and epithelial cells in the lungs, small intestine, and kidneys—showed enrichment of tissue remodeling pathways, including Angiogenesis, Coagulation, and Epithelial–Mesenchymal Transition.

Notably, some cell types—immune cells in the skin and epithelial and immune cells in the large intestine—showed enrichment of proliferation-related pathways. Inclusion of additional terms from the Reactome and Hallmark collections (Figure S2) revealed that activation of cell cycle pathways was accompanied by consistent enrichment of DNA damage response and telomere stress pathways (Reactome DNA Double-Strand Break Repair, Reactome G2/M DNA Damage Checkpoint, Reactome Telomere Stress-Induced Senescence, Reactome DNA Repair, Reactome Telomere Maintenance, Hallmark DNA Repair), a pattern broadly consistent with senescence induced by replicative exhaustion.

### 2.3. Upregulation of Antigen Processing and Presentation, Coupled with Activation of Inflammatory Pathways, Is a Hallmark of Senescent Cells Across Tissues

**Figure 6:**
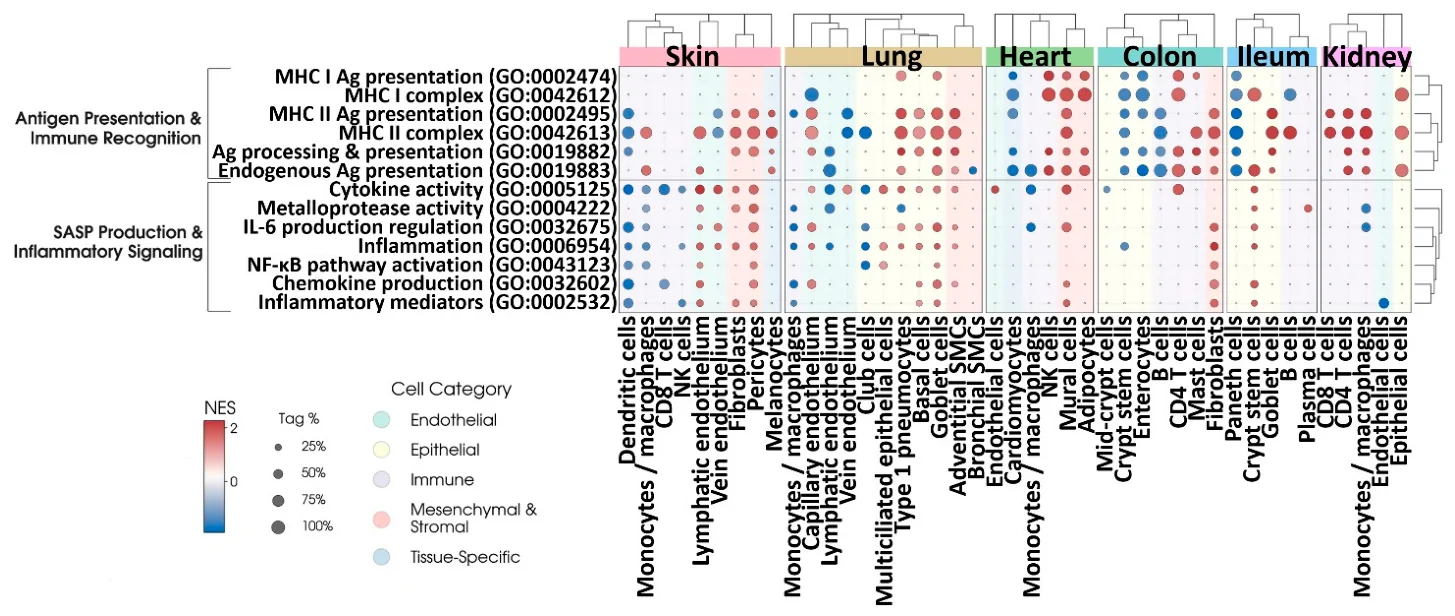
Landscape of antigen presentation and inflammatory signaling in senescent cells across different tissues and cell types. Dot plot showing enrichment of antigen presentation and inflammatory signaling pathways in senescent cells across different tissues. Color intensity represents normalized enrichment score (NES), and dot size indicates the percentage of genes from each term found in the cell type.

There is growing evidence that adaptive immunity plays a role in senescent cell clearance [37,38,39,40]. We therefore assessed the activation of antigen processing and presentation programs, as well as inflammatory signaling programs, in senescent cells across tissues (Figure 6). This analysis revealed transcriptomic patterns indicative of activated antigen processing and presentation in multiple cell types across tissues. In the skin and lungs, MHC-II-dependent antigen presentation was activated in non-canonical mesenchymal and endothelial cell populations—fibroblasts, pericytes, smooth muscle cells, and venous and lymphatic endothelial cells. MHC-II-dependent presentation was also upregulated in senescent fibroblasts and mast cells of the large intestine, goblet cells and B cells of the small intestine, mural cells of the heart (vascular smooth muscle cells and pericytes), and epithelial cells, CD4^+^ and CD8^+^ lymphocytes, and monocyte-macrophage lineage cells of the kidneys. In particular, pronounced activation of MHC-I-dependent antigen presentation was observed in senescent cells of certain tissues. Specifically, NK lymphocytes, mural cells, and adipocytes in the heart; CD4^+^ lymphocytes in the large intestine; and epithelial cells in the kidneys all showed enrichment of gene sets associated with antigen processing and presentation via MHC class I molecules.

Importantly, in most of the senescent cell types described above, upregulation of antigen presentation genes was accompanied by enrichment of pathways related to the production of pro-inflammatory cytokines and SASP factors. Specifically, inflammatory signaling genes were co-activated with antigen presentation genes in fibroblasts, pericytes, and venous and lymphatic endothelial cells of the skin; capillary endothelial cells, alveolar cells, adventitial smooth muscle cells, basal cells, and goblet cells of the lungs; fibroblasts of the large intestine; mural cells of the heart; and crypt stem cells of the small intestine.

### 2.4. Mapping Senescence Triggers and Mechanisms Across Diverse Tissues and Cell Types

**Figure 7:**
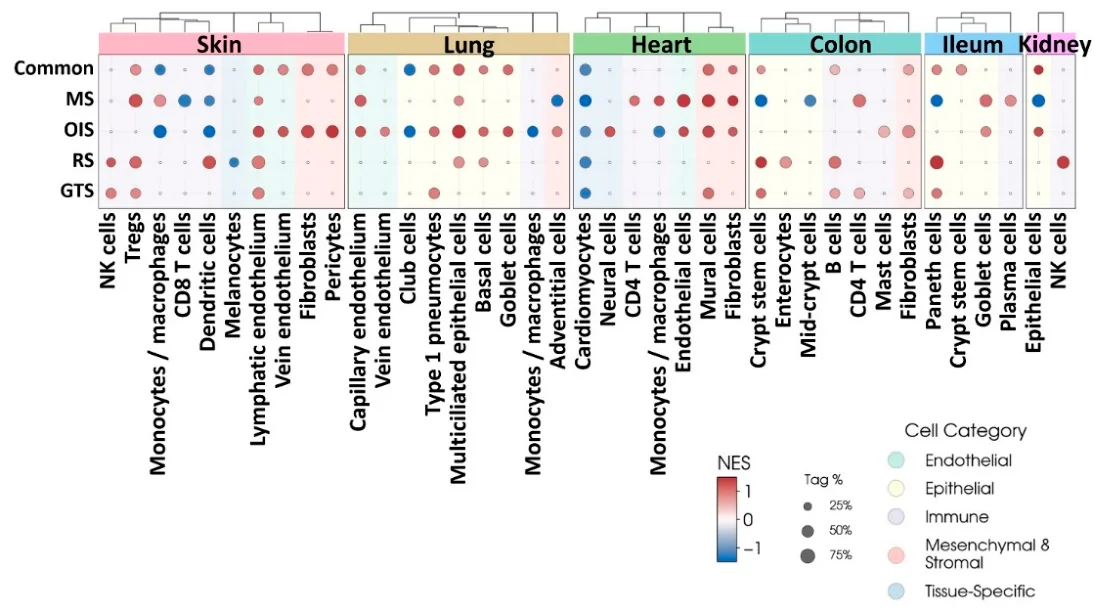
Cell type-specific enrichment of senescence programs. Dot plot showing normalized enrichment scores (NES) for senescence programs (Common, MS, OIS, RS, GTS) defined by conceptual grouping of related terms in senescent cell populations across different tissues. Color indicates NES (red: positive enrichment; blue: negative enrichment), and dot size represents the percentage of genes from each program detected in the cell type (gene sets were derived by taking the union of all genes from terms grouped into each senescence program).

Microenvironmental conditions and functional specialization are unique to each cell type. Consequently, distinct stressors differentially contribute to senescence induction in different cell types. To characterize the heterogeneity of senescence-associated transcriptional profiles, we curated 60 aging-related gene sets from multiple collections (see Section 4) and performed gene set enrichment analysis (Figure S2). We then tentatively classified these gene sets into several groups corresponding to distinct senescence trajectories: replicative (RS), genotoxic (GTS), oncogene-induced-like (OIS), and metabolism-associated (MS). We also defined a “general” group comprising terms that may be attributed to different senescence types depending on the biological context (Figure 7). It is important to emphasize that this classification is inherently provisional, given the absence of strict boundaries and substantial overlap among transcriptional programs of different senescence types across physiological contexts.

Our analysis revealed a degree of tissue specificity, with certain senescence programs predominating in particular tissues. In the skin, mesenchymal and endothelial cells were enriched for OIS genes and genes from the “Common” group. A similar pattern was observed in epithelial (with the exception of club cells), endothelial, and mesenchymal cell types of the lungs. Most cardiac cell types showed upregulation of MS genes and “Common” group genes. Enterocytes, B lymphocytes, and crypt stem cells of the large intestine, along with Paneth cells of the ileum, were enriched for genes associated with replicative exhaustion. At the same time, certain cell types displayed suppression of senescence-associated transcriptional programs. In particular, senescent cardiomyocytes identified by the algorithm exhibited pronounced downregulation of canonical senescence signatures and widely recognized aging markers. This finding was corroborated by SenMayo signature enrichment analysis, which revealed decreased activity of canonical senescence genes (Figure 3). Collectively, these data reflect the high heterogeneity of senescence states, in which cells across different tissues exhibit unique transcriptional profiles shaped by their specific microenvironment and function. Moreover, for certain cell types (e.g., cardiomyocytes), canonical senescence markers proved uninformative.

### 2.5. Senescent Cell-Driven Intercellular Signaling Across Tissues

**Figure 8:**
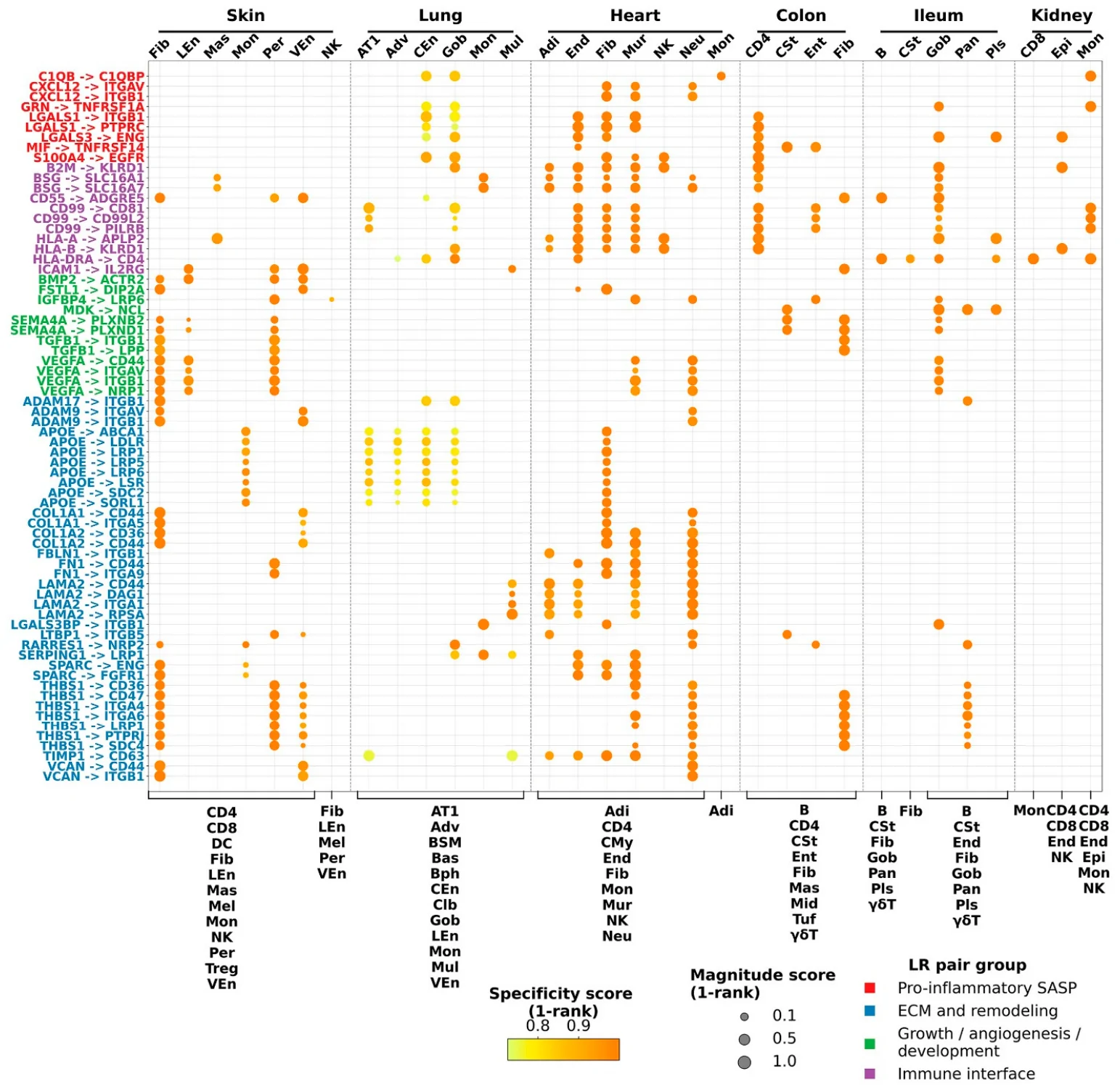
Cell type-specific ligand–receptor interaction analysis across tissues using LIANA. Dot plot showing enriched ligand–receptor (LR) pairs between senescent and neighboring cell types across six human tissues. LR pairs are grouped into four functional categories: pro-inflammatory SASP (red), ECM and remodeling (blue), growth/angiogenesis/development (green), and immune interface (purple). Each dot represents a specific LR interaction between ligand-expressing cells (top labels) and receptor-expressing cells (bottom labels) within each tissue. Dot color indicates specificity score, reflecting the selectivity of the interaction, while dot size represents magnitude score, indicating the strength of the interaction. Abbreviations: AT1—Type 1 pneumocytes, Adv—Adventitial cells, Adi—Adipocytes, B—B cells, Bas—Basal cells, Bph—Basophil, BSM—Bronchial SMCs, CD4—CD4 T cells, CD8—CD8 T cells, Cen—Capillary endothelium, Clb—Club cells, CMy—Cardiomyocytes, CSt—Crypt stem cells, DC—Dendritic cells, End—Endothelial cells, Ent—Enterocytes, Epi—Epithelial cells, Fib—Fibroblasts, Gob—Goblet cells, Len—Lymphatic endothelium, Mas—Mast cells, Mel—Melanocytes, Mid—Mid-crypt cells, Mon—Monocytes/macrophages, Mul—Multiciliated epithelial cells, Mur—Mural cells, NK—NK cells, Neu—Neural cells, Pan—Paneth cells, Per—Pericytes, Pls—Plasma cells, Treg—Tregs, Tuf—Tuft cell, Ven—Vein endothelium, γδT—γδ T cells.

**Figure 9:**
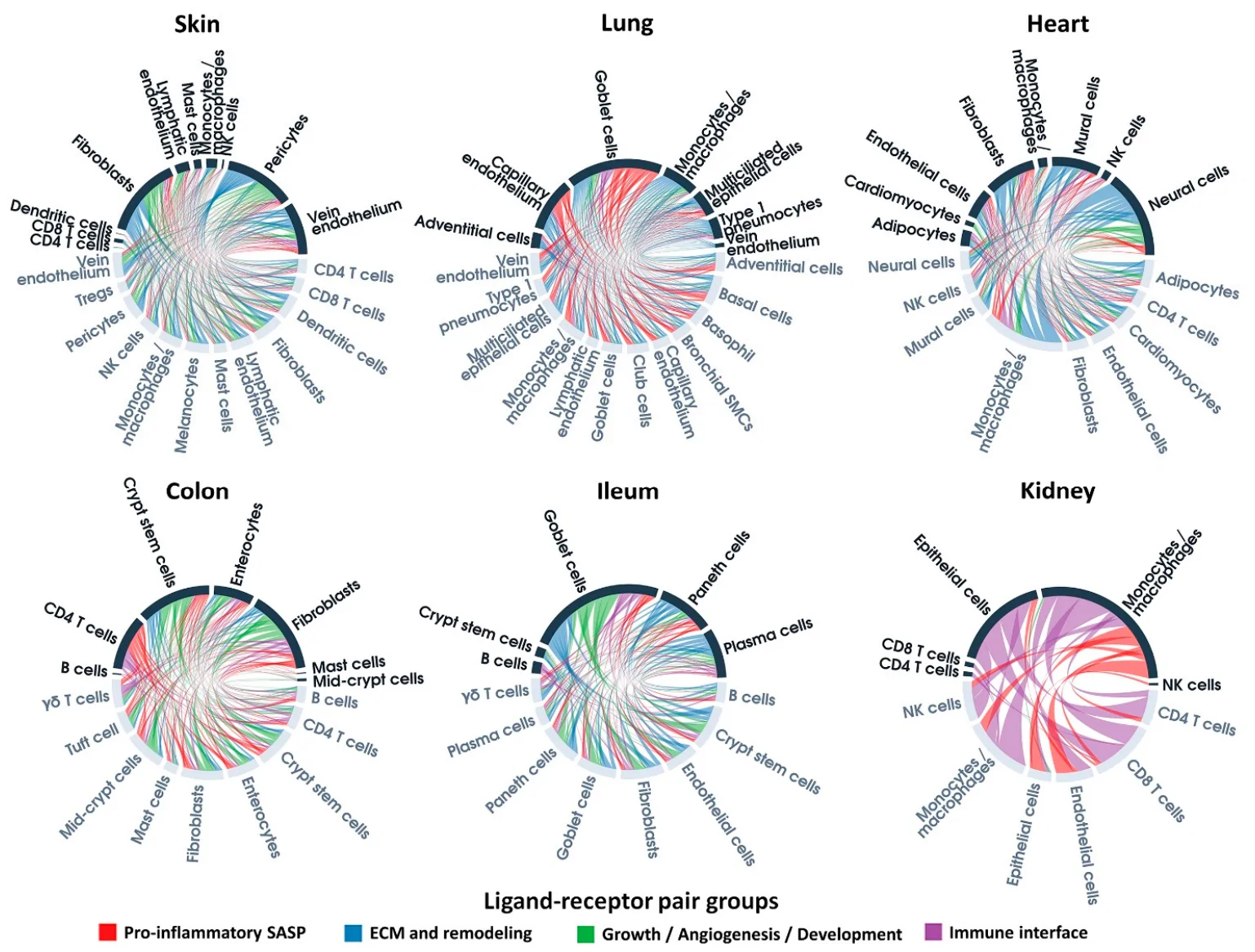
Chord diagrams depicting ligand-receptor interactions between senescent and neighboring cell types across six human tissues. Each circular diagram represents a tissue, with cell types arranged around the perimeter. Colored chords connect ligand-expressing (dark-colored) and receptor-expressing (light-colored) cell pairs, categorized by functional groups: pro-inflammatory SASP (red), ECM and remodeling (blue), immune interface (green), and growth/angiogenesis/development (purple).

Intercellular interactions were analyzed using the LIANA algorithm (see Section 4). A total of over 534,000 ligand–receptor interaction combinations were tested across all cell types in the dataset, 246,000 of which corresponded to interactions between senescent and non-senescent cells. For each interaction, LIANA computes composite significance metrics, including interaction strength and specificity. Among the tested interactions, we retained statistically significant pairs in which ligands were expressed by senescent cells and their cognate receptors by healthy target cells. The total number of significant interactions was 9829, of which the top 70 most prominent interactions with the highest strength and specificity scores were selected and visualized for each tissue (Figure 8). Interactions were further grouped into SASP categories: pro-inflammatory signals, extracellular matrix remodeling signals, angiogenesis-related signals, and immune-related signals (tissue-specific data for the top 100 strongest interactions and category lists are provided in Supplementary Figures S3A and S3B, respectively). Chord diagrams in Figure 9 illustrate significant interactions between senescent and non-senescent cells, revealing tissue-specific communication patterns and highlighting individual senescent cell types as dominant SASP signaling hubs.

In the skin, senescent fibroblasts, pericytes, and venous endothelial cells exerted the dominant influence, acting largely through extracellular matrix components (VCAN → ITGB1/CD44, COL1A1/COL1A2/COL4A1/COL4A2 → CD44), adhesion molecules (LAMC1/SELE → CD44, ICAM1 → IL2RA/IL2RG/CAV1), and the chemokine CCL2 → ACKR1. These signals targeted a broad range of non-senescent cell types, including various lymphocyte populations, fibroblasts, melanocytes, pericytes, resident macrophages, and endothelial cells (Figure 8).

In the lungs, pro-inflammatory interactions associated with SASP factors were notably upregulated. Senescent macrophages, goblet cells, and capillary endothelial cells emerged as the principal sources of inflammatory signaling. Key inflammatory mediators included acute-phase proteins (SERPING1 → SELP/LRP1, SAA1 → FPR1/CD36), calprotectin (S100A8/A9 → CD36/CD68/CD69/AGER), galectin interactions (LGALS3BP → ITGB1, LGALS3 → MCAM/ENG/PTPRC, LGALS1 → ITGB1/CD69), and inhibins (INHBA → TGFBR3/ENG/ACTR2). These signals primarily targeted endothelial cells and most immune cell populations (Figure 8).

In the heart, senescent fibroblasts and mural cells exhibited the highest signaling activity, with additional contributions from adipocytes, neutrophils, NK cells, and endothelial cells. These cells modulated the microenvironment through extracellular matrix remodeling factors (SPARC → ENG/FGFR1, THBS1 → CD36/CD47/ITGA6/LRP1, TIMP1 → CD63), galectins (LGALS1 → ITGB1/PTPRC, LGALS3 → ENG, LGALS3BP → ITGB1), angiogenic and chemotactic factors (VEGFA → CD44/ITGAV/ITGB1/NRP1, CXCL12 → ITGAV/ITGB1), and extracellular matrix components (COL1A1/COL1A2 → CD44, FN1 → CD44/ITGA9, LAMA2 → CD44/DAG1/ITGA1/RPSA, VCAN → CD44/ITGB1), alongside immune-modulatory axes (B2M → KLRD1, HLA-A → APLP2, HLA-B → KLRD1, HLA-DRA → CD4, MIF → TNFRSF14). (Figure 8).

In the large intestine, senescent fibroblasts exhibited the highest signaling activity, modulating the microenvironment through growth factors (TGFB1 → LPP/ITGB1/CXCR4), calcium-binding proteins (S100A4 → ERBB3/ERBB2/EGFR), thrombospondins (THBS1 → PTPRJ/LRP1/ITGA6/ITGA4), and MHC class I molecules (HLA-E/HLA-C/HLA-B → CD8A/CD8B/KLRD1). Senescent CD4^+^ T cells also displayed substantial activity via S100A4 and galectins (LGALS1 → PTPRC/CD69), as well as the HLA-B → KLRD1 and B2M → KLRD1/KLRC1/KLRC2 axes. Notably, senescent T lymphocytes and fibroblasts actively signaled to γδ T cells via MHC class I molecules (HLA-A/B/C/E → CD8A/CD8B) and through ligands of the inhibitory CD94/NKG2A–NKG2C receptor complex (HLA-B → KLRD1, B2M → KLRD1/KLRC1/KLRC2) (Figure 8).

In the ileum, senescent goblet cells exerted the greatest influence, acting through the galectin family (LGALS3 → MCAM/LAG3/ENG, LGALS9 → PTPRK/PTPRC, LGALS3BP → ITGB1), laminins (LAMC2/LAMB3/LAMA3 → CD44), the MDK → TSPAN1/NCL/LRP1 axis, and apolipoproteins (APOC3/APOB/APOA4/APOA1 → LRP1/LDLR/MTTP/ENO1/ABCA1). Interestingly, senescent B cells actively engaged the inhibitory receptor LAG3 on γδ T cells via MHC class II molecules (HLA-DRB1/HLA-DRA/HLA-DPB1 → LAG3) (Figure 8).

In kidney tissue, senescent epithelial cells and monocytes/macrophages exerted the most prominent effects. Specifically, senescent monocytes/macrophages exhibited a broad repertoire of interactions, targeting the inhibitory receptor LAG3 on CD8^+^ T lymphocytes and the CD4 co-receptor on non-senescent monocytes/macrophages via MHC class II molecules (HLA-DRB1, HLA-DRA, HLA-DQB1, HLA-DQA1, HLA-DPB1, and HLA-DPA1). Furthermore, these monocytes/macrophages signaled via the GRN ligand to the TNFRSF1A/B receptors, and via CD14 to the ITGB2, ITGA4, and ITGB1 receptors expressed on CD4^+^ and CD8^+^ T lymphocytes, NK cells, and healthy monocytes/macrophages. In turn, senescent epithelial cells targeted NK cells via the HLA-B → KLRD1 and B2M → KLRD1/KLRC1/KLRC2 axes (Figure 8).

These data demonstrate that senescent cells actively shape the tissue microenvironment through complex intercellular communication networks. During aging, each tissue develops unique signaling patterns that reflect its specific physiology and distinct vulnerabilities.

## 3. Discussion

De novo reproduction of the SenePy algorithm yielded unique aging signatures, which were used to compute a senescence score for each cell within a given cell type [35]. Overall, the algorithm’s accuracy was corroborated by the significant enrichment of genes from the consensus SenMayo senescence signature in nearly all examined senescent cell types across tissues (compared to non-senescent cells of the corresponding types) [36]. Notably, however, two senescent cell types—cardiomyocytes and skin dendritic cells—exhibited the opposite trend, showing suppression of the SenMayo signature (Figure 3). This may reflect both the specific physiology of these cell types and the limited universality of the SenMayo signature. For instance, mature active dendritic cells (DCs) upregulate co-stimulatory molecules, chemokine receptors, and pro-inflammatory cytokines, whereas senescent DCs are characterized by transcriptional downregulation and reduced activation capacity. Consequently, the aggregate expression score of pro-inflammatory genes (including SenMayo SASP genes) may be higher in healthy, active DCs than in non-functional senescent DCs [41,42]. In turn, the suppression of the SenMayo signature in senescent cardiomyocytes may stem from fundamental physiological differences in the aging of proliferating versus post-mitotic cells [43]. The SenMayo signature was derived primarily from transcriptomic data of mitotic mesenchymal cells, which explains the predominance of classic proliferative arrest markers and SASP genes typical of myeloid, stromal, and endothelial cells in its core [44]. Cardiomyocytes, however, are post-mitotic cells with an atypical secretory phenotype, and apparently most classic SenMayo signature genes are epigenetically repressed in them [45,46]. Furthermore, the dominance of structural and contractile protein transcripts in cardiomyocytes—which is exacerbated during age-related hypertrophy against a backdrop of stress-induced global transcriptional decline—may shift SenMayo genes to the tail of the ranked list. This explains the artifact of signature suppression compared to healthy cardiomyocytes. To test this hypothesis, we curated a cardio-selective senescence signature based on the published literature (BMP10, GDF15, IGFBP7, SERPINE1, CDKN1A, CDKN2A, TP53, GADD45G, GADD45A, PTCHD4, SESN1, SESN2, FDXR, BBC3, MDM2, TP53INP1) [47,48,49,50,51,52]. Validation of this signature on our data revealed significantly higher AUCell scores in cardiomyocytes classified as senescent by SenePy, further confirming the algorithm’s reliability for in vivo data (Figure S4). Notably, SenePy was specifically designed to overcome the limitations of universal senescence signatures: its developers demonstrated that the algorithm more accurately identifies transcriptomic patterns of aging cells across diverse cell types [35], whereas SenMayo relies predominantly on classic SASP genes and proliferative arrest markers characteristic of mitotically active populations [36,44].

Aging proceeds unevenly across organs and tissues due to physiological and environmental differences. Investigation of the age-related dynamics of senescent cell accumulation across organs revealed pronounced heterogeneity (Figure 4) [53]. Interestingly, the greatest number of significant correlations was observed predominantly in barrier tissues—the skin, lungs, and intestine. This likely reflects, on the one hand, the contribution of exogenous stressors to senescent cell accumulation, and on the other, differences in the proliferative activity and turnover rates of epithelial and stromal compartments across organs. The involvement of tissue-resident myeloid and lymphoid cells in the aging process reflects not only the establishment of an inflammaging milieu but also, presumably, an age-related decline in the immune system’s ability to clear senescent cells [38,54]. In some cell types, significant correlations were absent, although the overall trend toward an increased senescent cell fraction persisted. This is attributable to high donor variability as well as technical factors, such as differences in sequencing depth and information loss during batch effect correction.

Importantly, our analysis of senescent cell fractions does not account for age-related shifts in overall tissue composition, such as the decrease in the absolute number of fibroblasts in aging skin or the increase in regulatory T cells. The latter may reflect the emergence of peripheral tolerance to senescence-associated antigens during aging, which could also contribute to the reduced immune clearance of senescent cells [54,55]. Despite growing indirect evidence, the question of whether adaptive immunity is directly involved in controlling senescent cell abundance across tissues remains open [39]. We therefore assessed the activity of transcriptional programs related to antigen presentation by senescent cells, as well as their production of inflammatory factors that recruit immune cells (Figure 6). Despite the pronounced heterogeneity of senescent cell transcriptomic programs, reflecting the diversity of inducing stimuli (DNA damage response, replicative exhaustion, metabolic and oxidative stress; Figure 5), enrichment analysis revealed a common feature associated with the activation of antigen presentation via MHC-I- and MHC-II-dependent mechanisms [56,57,58,59]. Coupled with increased pro-inflammatory signaling (Figure 6), this indicates the potential immunogenicity of senescent cells and aligns with the concept of their clearance involving adaptive immunity.

Collectively, the GSEA data (Figure 5) point to the high heterogeneity of senescent cells across different cell types and tissues. Thus, senescence is characterized by a family of related states, with the specific features of each dictated by cell origin and tissue context. Mapping of enriched senescence-associated gene sets revealed that the dominant trajectory is likely consistent with tissue-specific physiology (Figure 7). For instance, the metabolic stress transcriptional program predominated in the heart [60,61]; in the intestine, alongside replicative exhaustion of enterocytes and crypt stem cells [62,63,64,65], there was activation of the genotoxic stress program in fibroblasts and immune cells; and in the skin and lungs, oncogene-induced-like senescence was activated [16,66,67]. We note that the categorization of enriched gene sets into discrete groups (Figure S2) is inherently provisional, as these transcriptional programs overlap substantially [68]. Nevertheless, this approach demonstrates that senescent cell heterogeneity is context-dependent rather than random.

Alongside the activation of DDR, cell cycle arrest, and metabolic reprogramming programs [12,13,14,15,16], senescent cells of many types showed enrichment of signatures associated with the production of inflammatory SASP factors [10,15,19]. At the transcriptomic level, the main SASP producers were fibroblasts and endothelial cells of the skin and intestine, epithelial cells of the lungs, and endothelial and mural cells of the heart (Figure 5 and Figure 6). This reflects the contribution of senescent cell accumulation to the low-grade systemic inflammation of old age, which is associated with an increased risk of all age-related diseases [69,70]. In this context, it is worth noting that the transition to a senescent state may be driven not only by external adverse exposures but also by the local microenvironment [68]. To investigate the impact of senescent cells on neighboring microenvironmental cells across tissues, we performed intercellular interaction analysis using the LIANA algorithm [71], focusing on outgoing signals from senescent cells (Figure 8). The analysis demonstrated that these cells profoundly influence the microenvironment, with specific signal groups predominating in different tissues (Figure 8 and Figure 9). Extracellular matrix remodeling and tissue development signals predominated in the skin due to the activity of senescent fibroblasts and pericytes, and in the heart via fibroblasts, mural cells, and neural cells. Pro-inflammatory signals were prominent in the lungs, large intestine, and kidneys. Their primary sources were goblet cells and endothelial cells in the lungs, fibroblasts and CD4^+^ lymphocytes in the intestine, and monocyte–macrophage lineage cells in the kidneys. Additionally, immune signaling mediated primarily by senescent epithelial and monocyte-macrophage lineage cells was pronounced in the kidneys. Importantly, a wide range of paracrine senescence mediators was identified among the outgoing signals, confirming the ability of senescent cells to induce secondary senescence in the microenvironment [8,11,68]. Of particular interest is the inhibitory effect of senescent cells on various leukocyte populations observed across all tissues. Specifically, senescent cells exhibited transcriptional signatures of active immune evasion, protecting against NK and CD8^+^ T cell-mediated clearance. This is mediated through the upregulation of MHC-I molecules (including non-classical HLA-E) and B2M, which engage the KLRD1(CD94)/KLRC1(NKG2A) inhibitory complex on NK cells, as well as through the expression of MHC-II and galectin-3, which bind the LAG3 inhibitory receptor on CD4^+^, CD8^+^, and γδ T lymphocytes [72,73,74,75]. Furthermore, the increased expression of galectins LGALS1 and LGALS9 by senescent cells promotes the apoptosis, anergy, and functional exhaustion of activated lymphocytes via interactions with TIM-3 (for LGALS9) and receptors such as PTPRC (CD45) and CD69 (for LGALS1) [76,77,78]. Additional immunosuppressive signaling axes included the integrin-mediated activation of latent TGFB1 (e.g., via ITGB1), which drives regulatory T cell expansion and effector T cell suppression [79]; GRN binding to TNFRSF1A, antagonizing pro-inflammatory TNF signaling; and the THBS1-CD47 axis, which transmits a “don’t eat me” signal to macrophages to suppress phagocytosis (Figure 8 and Figure S3A) [80,81]. Because senescent cell clearance relies heavily on NK cells and apparently cytotoxic lymphocytes, the simultaneous upregulation of these inhibitory checkpoints represents a coordinated mechanism of immune evasion, mirroring strategies extensively characterized in tumor immune escape [71,72,73,74,75,76,77,78,79,80]. Therapeutic blockade of these checkpoints holds significant potential to restore immune-mediated clearance of senescent cells, representing a promising avenue for next-generation senotherapies.

In the context of developing modern senotherapeutics, the high transcriptional heterogeneity of senescent cells identified in organs with distinct physiology underscores the need for more targeted strategies. Detailed mapping of the senescent landscape enables the distinction between “physiological” senescent cells, which perform reparative and structural functions, and pathological pro-inflammatory subpopulations [9]. The total elimination of senescent cells carries the risk of disrupting tissue homeostasis, inducing systemic toxicity, and triggering excessive inflammatory responses [82]. Therefore, targeted intervention directed at specific senescent cell populations within a given organ—even if not the most numerous, but rather the most pro-inflammatory and pathogenic—appears to be the most promising approach. Furthermore, immunological approaches, particularly the development of senolytic vaccines [39], are of significant interest in this context, as they hold the potential for the safe elimination of specific senescent cell populations.

This study utilized publicly available scRNA-seq data encompassing different experimental series, as well as diverse tissue dissociation and sample preparation protocols. It should be noted that such heterogeneity introduces technical variability, requiring rigorous computational correction and caution in result interpretation. Different platforms, dissociation protocols, and sample acquisition conditions underlie batch effects. Moreover, various stresses during sample preparation have a substantial impact—for example, enzymatic tissue dissociation can trigger a stress response that mimics a senescent profile [83,84]. Furthermore, sequencing depth greatly affects senescence signature enrichment estimates: SASP genes and core senescence-associated genes CDKN2A and CDKN1A have low expression and often fall below the detection threshold in scRNA-seq data [44,85]. Additionally, senescence signatures are not absolutely specific: SASP genes overlap with inflammatory response genes, DDR is induced by any DNA damage, and metabolic senescence signature genes are activated during hypoxia and energy imbalance. Therefore, working with such data requires balanced analysis, and the conclusions necessitate experimental validation.

## 4. Material and Methods

For this analysis, we collected publicly available scRNA-seq data from healthy donors aged 15 to 88 years. For skin, lungs, small intestine, and colon, we obtained raw sequencing data and generated count matrices with 10× Genomics Cell Ranger v.9.0.1. For kidney and heart, published count matrices were used because raw data were unavailable. All count matrices and associated metadata were processed with Scanpy [86]. Quality control involved removing cells with fewer than 500 detected genes or UMIs, cells lying outside ±5 median absolute deviations (MAD) for these metrics, cells with a mitochondrial gene expression fraction exceeding 0.4 or 4 MAD, and doublets identified by Scrublet [87]. After filtering, the final dataset comprised approximately 1.5 million cells from six tissues and 177 donors (Supplementary Figure S1 and Table S1). Within each tissue, we integrated samples using single-cell Variational Inference (scVI) and annotated cell types by transferring labels from matched tissue data in the Tabula Sapiens atlas with scANVI (single-cell Annotation using Variational Inference) [88,89]. We then manually inspected the resulting annotations for each tissue. To correct for differences in sequencing depth across samples from different sources, we downsampled the original data to a comparable number of detected genes and derived senescent-cell signatures from these downsampled profiles. All downstream analyses were performed on the original full-depth data without additional downsampling (Figure 1).

### 4.1. Identification of Cell Type-Specific Senescence Signatures

Potential cellular senescence signatures were identified using an algorithm based on age-related gene expression dynamics and co-expression network analysis of scRNA-seq data. This algorithm was previously described in [35] and validated through several independent biological assays.

The data were stratified by tissue and cell type. Cell populations comprising at least 100 cells in both age cohorts—including young (≤31 years) and older (≥61 years) donors—were selected for analysis. For each gene, a Gene Age-Dynamic (GAD) score was calculated, taking into account the fraction of positive cells in young donors (≤2%), the increase in this fraction among older donors (≥20%), and the slope of the linear regression of expression against age. To filter out noise, these values were compared against a null distribution generated through permutation testing.

Next, the expression matrix of significant dynamic genes was binarized, with 0 indicating no detectable expression and 1 indicating expression supported by at least one UMI. Pearson correlations were then computed for all gene pairs. Statistically significant correlations were used to build gene co-expression graphs, and the Leiden algorithm was applied to extract dense clusters, thereby excluding sparsely connected genes [90]. The resulting stable gene clusters formed 64 signatures, one per annotated cell type, across the analyzed tissues.

### 4.2. Senescence Scoring and Binarization

Senescence scores for individual cells were calculated using the method implemented in scanpy.score_genes. To control for background expression, all genes in the dataset were divided into 25 bins based on their mean expression levels. For each gene in the target signature, 50 control background genes were randomly sampled from the corresponding expression bin. The original expression matrix was then binarized to represent the discrete activity state of the senescence program. Signature gene values were multiplied by their individual importance weights, defined by gene centrality (i.e., the number of connections within the signature’s co-expression graph). Finally, the overall senescence score (sen_score) for each cell was computed as the difference between the weighted average expression of the target signature genes and the mean expression of the control background gene set:

$${Score}_{i}=\;\bar{Y_{i,S}}-\;\bar{Y_{i,BG}},\;\;for\;i\in all\;cells;$$

where ${Score}_{i}$ is the SenePy score for cell *i*, S denotes the gene signature, and BG denotes the background gene set.

Cell types were included in the analysis if they contained data from at least three donors and at least 100 cells per donor. To identify senescent cells (Sc), per-cell senescence score ${Score}_{i}$ distributions were obtained. The resulting scores were centered by subtracting the median score for each donor within each cell type. An adaptive binarization threshold based on two-component Gaussian mixture models (GMMs) was then applied to the centered data. Cells with scores exceeding the identified threshold were classified as senescent, whereas cells with scores below the threshold were classified as non-senescent (NSc).

### 4.3. Determination of Adaptive Binarization Thresholds Using GMMs

We used a two-stage adaptive binarization algorithm based on Gaussian mixture models (GMMs) to separate cell populations into non-senescent (NSc) and senescent (Sc) fractions based on continuous scores. In the first stage, the observed score distribution was fitted with a two-component GMM initialized using the 25th and 75th percentiles of the sample. Distribution bimodality was then assessed using the Fisher separability metric:

$$S=\frac{\left|\mu_{1}-\mu_{2}\right|}{\sqrt{0.5\times (\sigma_{1}^{2}+\sigma_{2}^{2})}};$$

where µ and $\sigma_{i}^{2}$ denote the expected values and variances of the identified components, respectively. If peak separation was insufficient (S < 3), a kernel density estimate (KDE) was used to locate the global mode of the distribution. The left “noise” tail (*x < mode—*0.8*σ*) was removed from the sample, and subsequent GMM training was performed on the truncated dataset to minimize parameter bias. If S > 3, the model was trained on the full dataset. Before training, extreme outliers were truncated at the 99.9th percentile.

The final cutoff threshold was determined using an iterative procedure for fitting a two-component GMM ($n_{init\;}=5$, max _iter=100). To stabilize convergence under class imbalance, an a priori target proportion of the senescent component (0.1) was specified. If component separability was unsatisfactory (S < 0.5), the algorithm performed up to 10 restarts with stepwise changes in the initialization parameters. Cells were classified based on posterior probabilities of belonging to the “high” (senescent) component. The binarization threshold was mathematically defined as the minimum score at which the posterior probability satisfied P_(High Comp)_ ≥ 0.6.

To prevent overestimation of the target class fraction due to false positives (for example, in samples with unimodal distributions), a conservative safeguard was implemented: if the estimated threshold assigned more than 70% of cells to the senescent class, the final threshold was forcibly recomputed as the 85th percentile of the original distribution.

### 4.4. Functional Analysis

To assess the extent of cellular senescence in different cell types, we applied AUCell, a gene-signature enrichment analysis method from the decoupler package (v. 2.1.4). The validated SenMayo signature, consisting of 125 conserved marker genes of cellular senescence, was used as the reference profile. The AUCell algorithm is independent of data normalization units and batch effects because it ranks genes within each individual cell [91]. For each cell, all expressed genes were ranked in descending order of expression level. The enrichment score was computed as the area under the recovery curve (AUC) of the ranked gene list. The resulting AUC values reflect the proportion of genes in the SenMayo signature that are highly expressed in a given cell. Statistical comparisons of AUC scores between Sc and NSc cells were performed using the Wilcoxon test with Benjamini–Hochberg correction. Effect sizes were estimated using the rank-biserial correlation coefficient.

### 4.5. Differential Gene Expression Analysis (DGEA)

Senescence-associated genes were identified through differential gene expression analysis (DGEA) for each cell type within the corresponding tissue. To generate pseudobulk samples, groups of at least 10 cells were aggregated for each cell type. Cell types containing data from at least three donors were included in the analysis. The analysis was performed using edgeR (v. 3.36.0). For each population, the raw count matrix was filtered to remove lowly expressed genes using filter_by_expr. Library composition was normalized using the trimmed mean of M-values (TMM) method, with individual normalization factors calculated for each sample.

To estimate the effect of cellular senescence while correcting for inter-individual variability, generalized linear models (GLMs) were used. For each cell type, a paired design matrix was constructed using the following linear formula: Y~donor_id+sen_group, where the donor_id covariate served as a blocking factor to account for inter-individual variability, and sen_group represented the target cell status (Sc vs. NSc). Common and trended dispersions were estimated using estimateDisp. Model parameter estimation and differential expression testing were performed using the GLM quasi-likelihood F-test via glmQLFit and glmQLFTest. This approach provides stringent and conservative control of the false discovery rate (FDR) by accounting for uncertainty in dispersion estimation for each gene.

### 4.6. Gene Set Enrichment Analysis (GSEA)

For biological interpretation of the differential expression results and identification of functional differences between Sc and NSc cells, pre-ranked GSEA was performed separately for each cell type. Full gene lists ranked in descending order of log_2_ fold-change (log_2_FC) values obtained from DGEA with edgeR were used as input. The analysis was performed using gseapy (v. 1.2.1).

To provide a general description of metabolic and signaling rearrangements, the standard Hallmark collection from the Molecular Signatures Database (MSigDB) was used [92]. To characterize the molecular mechanisms of senescence in detail, Gene Ontology (GO), MSigDB, and Reactome terms corresponding to the key senescence types—metabolic (MS), oncogene-induced-like (OIS), genotoxic (GTS), and replicative (RS)—were manually curated [93,94]. For each senescence type, a unified signature was generated by combining unique genes from the corresponding terms. In addition, specialized gene sets associated with secretion of SASP factors (Senescence-Associated Secretory Phenotype) and antigen presentation mechanisms were included.

Statistical significance of pathway enrichment was assessed by permutation testing. A biological pathway or signature was considered significantly altered if the FDR-adjusted *p*-value was <0.05 and the absolute normalized enrichment score was greater than 1 (|NES| > 1).

### 4.7. Ligand-Receptor Interaction Analysis

Ligand-receptor interactions mediated by SASP-associated factors were analyzed within tissues using LIANA+ [f] (v.1.8.0) [71]. The rank_aggregate() function was used to integrate scores obtained from different methods. We analyzed interactions in which Sc cells were ligand sources and NSc cells were targets. From all obtained interactions, we selected those whose ligands corresponded to differentially expressed genes in senescent cells and were classified as SASP factors. Next, we retained interactions in which the ligand was detected in more than 20% of cells of the corresponding cell type, the receptor was detected in more than 5% of cells, and the interaction magnitude rank was <0.25 (i.e., the final list included the top 25% of strongest interactions). Interaction strength was assessed using magnitude_rank; values closer to 0 indicate a higher consensus interaction rank.

## 5. Conclusions

In this study, we analyzed publicly available scRNA-seq data from various tissues obtained from 177 apparently healthy donors aged 15 to 88 years. In total, after applying quality control filters, approximately 1.5 million cells from six tissues were included in the analysis. Implementation of the SenePy algorithm involved analyzing age-related gene expression dynamics, as well as constructing and clustering gene co-expression networks. This enabled us to identify cellular aging signatures for 64 cell types. These marker gene sets served as the basis for identifying senescent cell fractions and their subsequent functional characterization. As a result, we obtained comprehensive data on the dynamics of senescent cell accumulation across tissues and on their transcriptional heterogeneity. Analysis of generalized terms reflecting various aging triggers suggested that the observed transcriptional heterogeneity is not random but is instead linked to the physiological characteristics of a specific cell type and tissue. Importantly, despite the simultaneous enrichment of antigen presentation and inflammatory signaling pathways, senescent cells in all examined tissues demonstrated a pronounced ability to evade immune surveillance through the expression of a broad spectrum of immune checkpoint inhibitors. This mechanism may underlie the age-related decline in the immune system’s efficiency in clearing senescent cells, and targeting these pathways holds potential for the development of novel senolytic strategies.

## Data Availability

Publicly available datasets were analyzed in this study. The analysis code, processed data, and specific Gene Expression Omnibus (GEO) accession numbers (GSE identifiers) required to access the original single-cell RNA sequencing (scRNA-seq) datasets are available in the following open-access repository: https://github.com/estakathersun/sen_heterogeneity_paper (accessed on 16 September 2026). No new primary data were generated during this study.

## Supplementary Materials

The following supporting information can be downloaded at: https://www.mdpi.com/article/10.3390/ijms27188416/s1.
