# Supplementary material for: Senescent Cell Heterogeneity: Tissue-Dependent Signatures and Age Dynamics Revealed by Human scRNA-Seq Data
Source: Int J Mol Sci. 2026 Sep 21;27(18):8416. doi: 10.3390/ijms27188416 (PMC13607090; doi:10.3390/ijms27188416)
Supplement: Supplementary file 1 [file ijms-27-08416-s001.zip › Supplementary_Figures S1-S4.pdf]

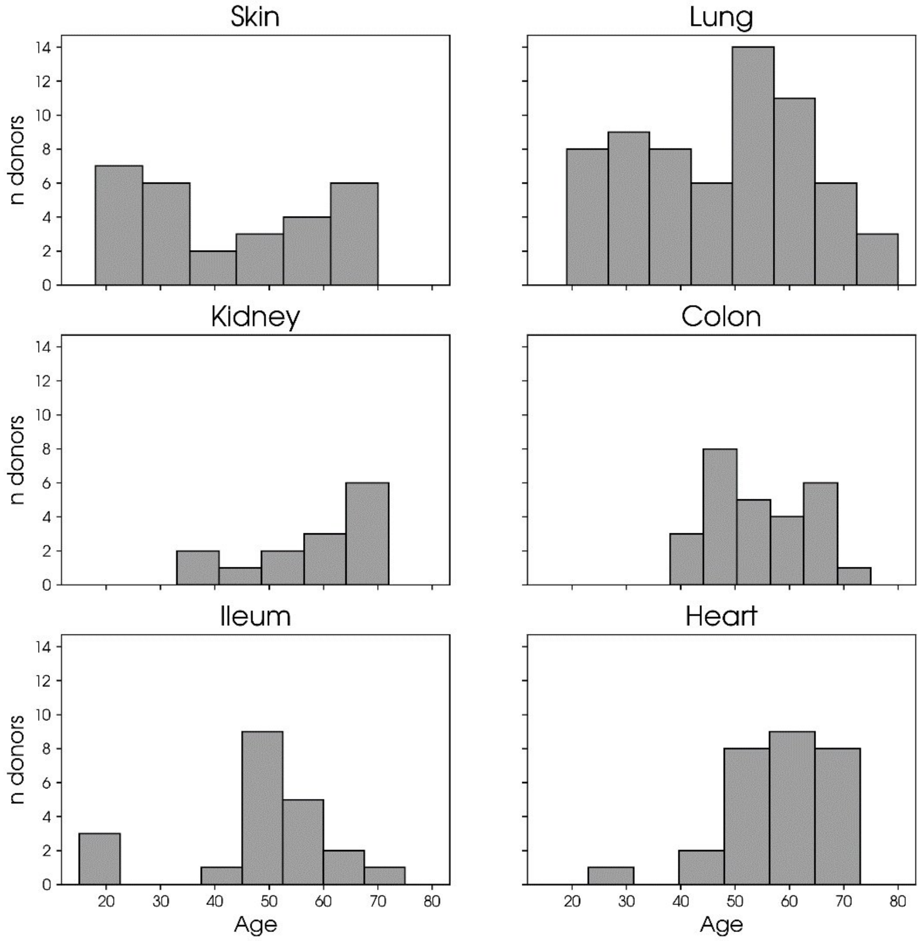

Figure S1. Age distribution of donors for each tissue.

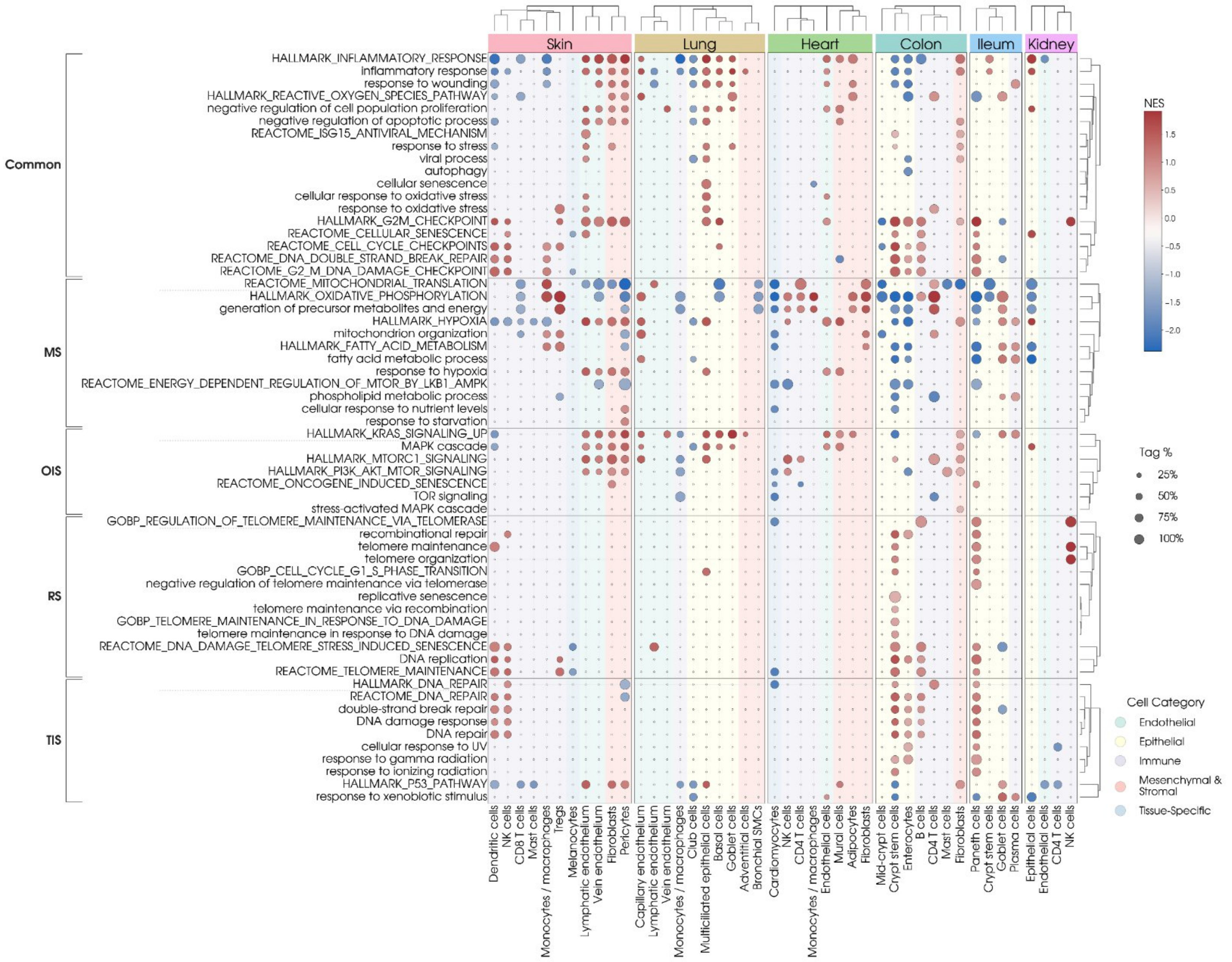

Figure S2. GSEA of senescent versus non-senescent cells. Dot color indicates the normalized enrichment score (NES; red: enriched, blue: suppressed). Dot size represents the proportion of genes from each gene set forming the leading-edge subset that contributes to the enrichment signal. Pathways are grouped according to the type of stress triggering the senescence program (MS, metabolic stress; OIS, oncogene-induced stress; RS, replicative stress; TIS, therapy-induced stress, also known as genotoxic stress, GTS).

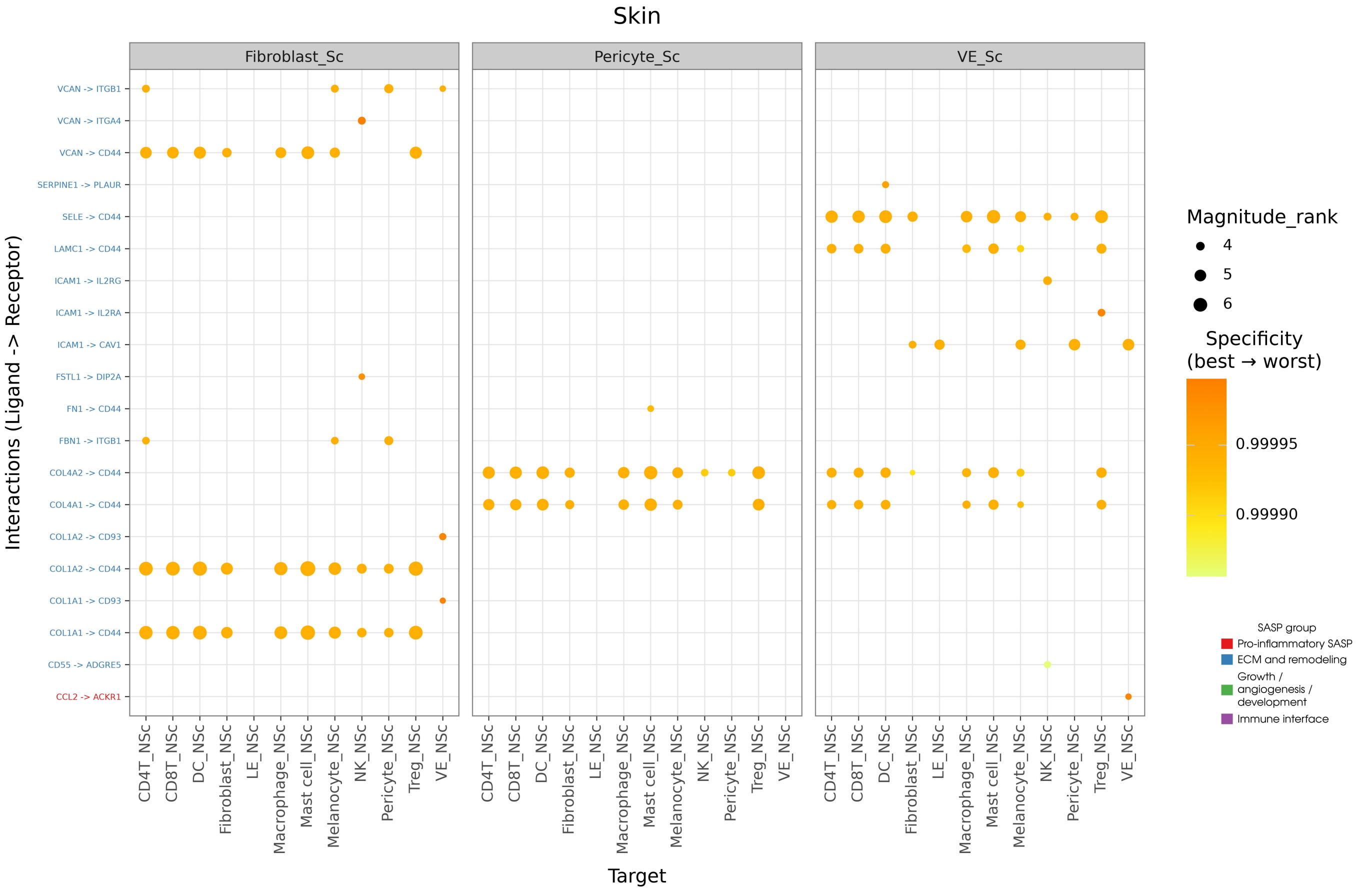

Figure S3A. Top 100 strongest interactions between Sc and NSc cells in Skin. For visualization, magnitude_rank and specificity_rank values were inverted, such that higher values indicate greater interaction strength and specificity, respectively.

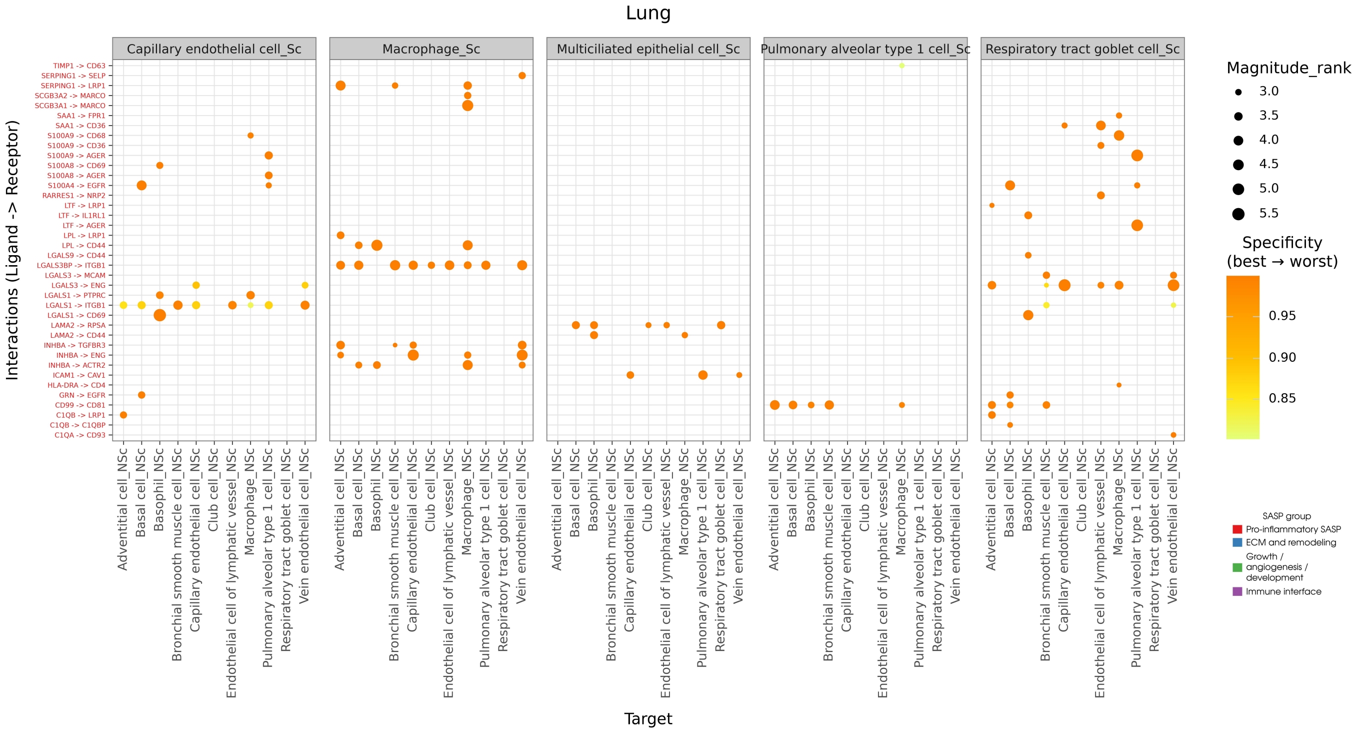

Figure S3B. Top 100 strongest interactions between Sc and NSc cells in Lung. For visualization, magnitude_rank and specificity_rank values were inverted, such that higher values indicate greater interaction strength and specificity, respectively.

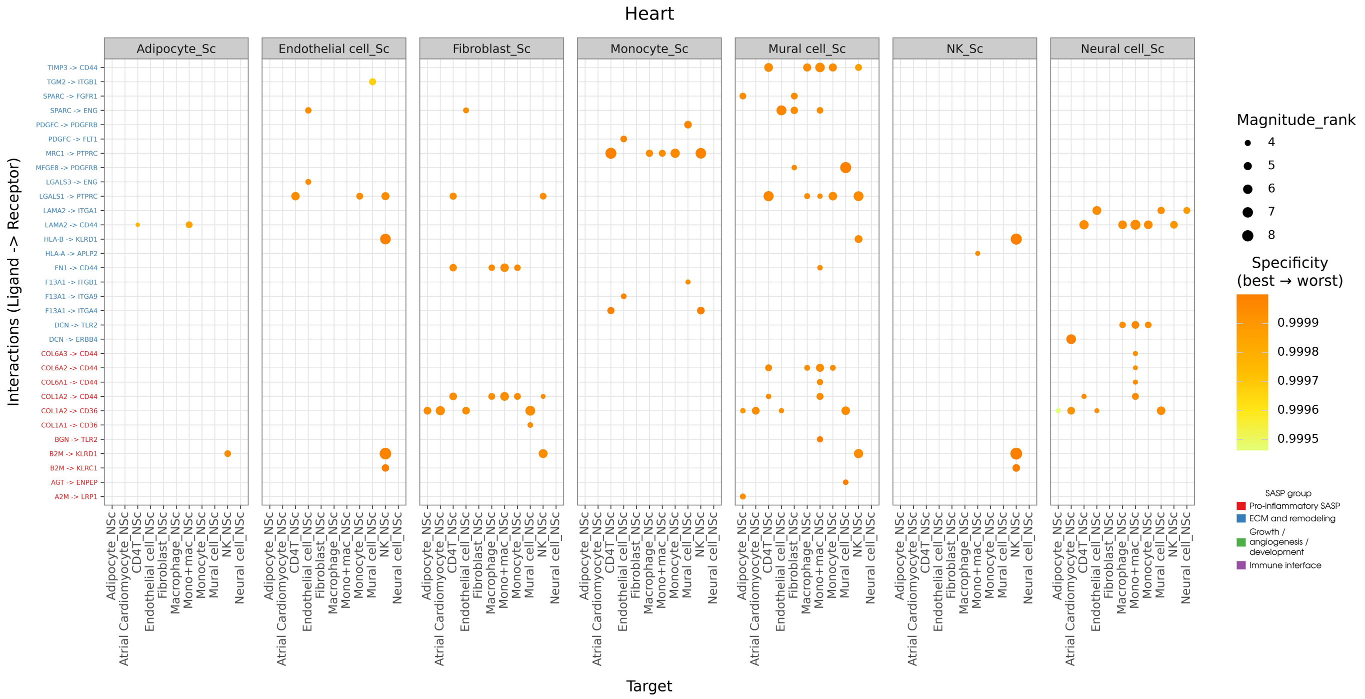

Figure S3C. Top 100 strongest interactions between Sc and NSc cells in Heart. For visualization, magnitude_rank and specificity_rank values were inverted, such that higher values indicate greater interaction strength and specificity, respectively.

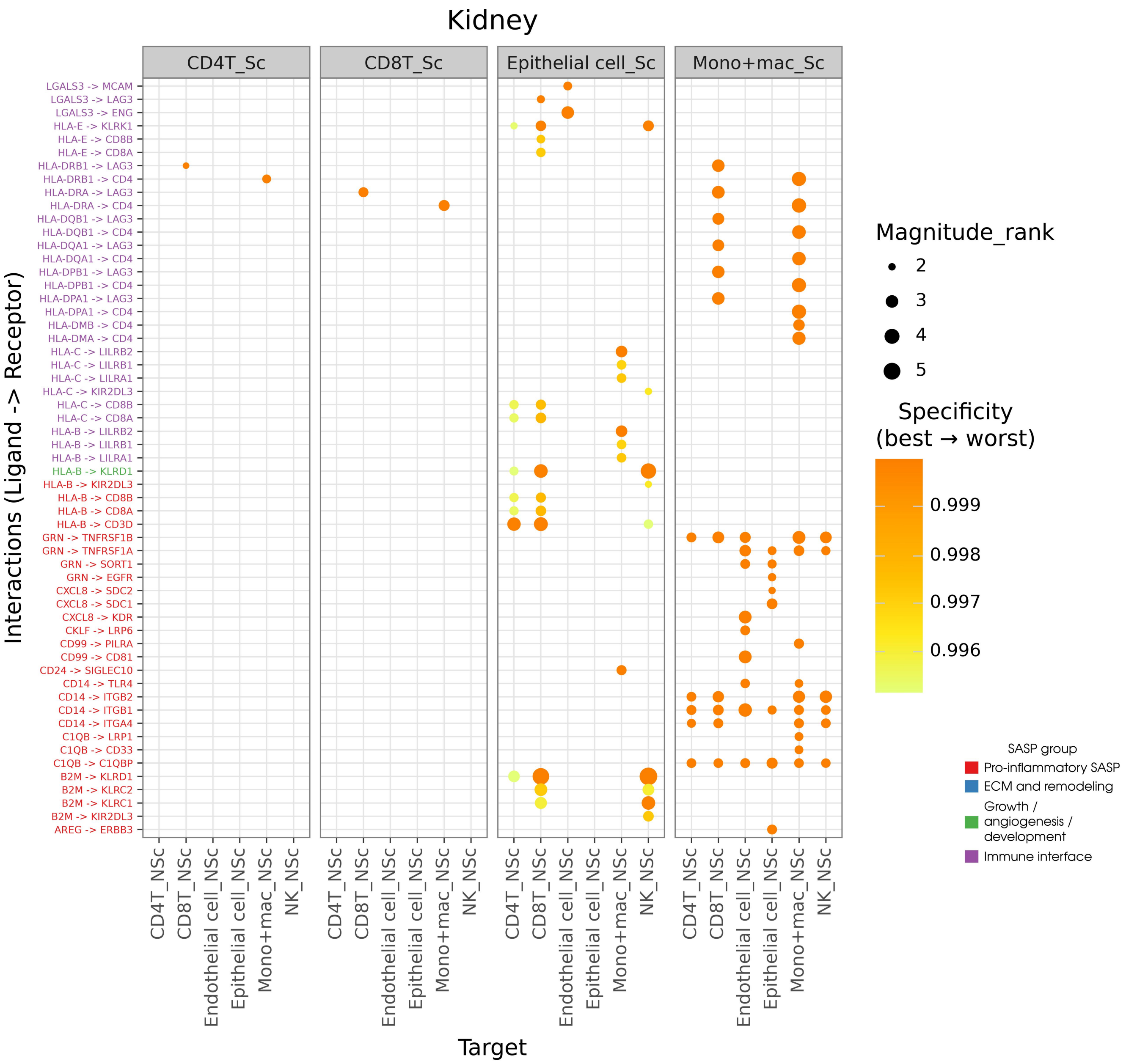

Figure S3D. Top 100 strongest interactions between Sc and NSc cells in Kidney. For visualization, magnitude_rank and specificity_rank values were inverted, such that higher values indicate greater interaction strength and specificity, respectively.

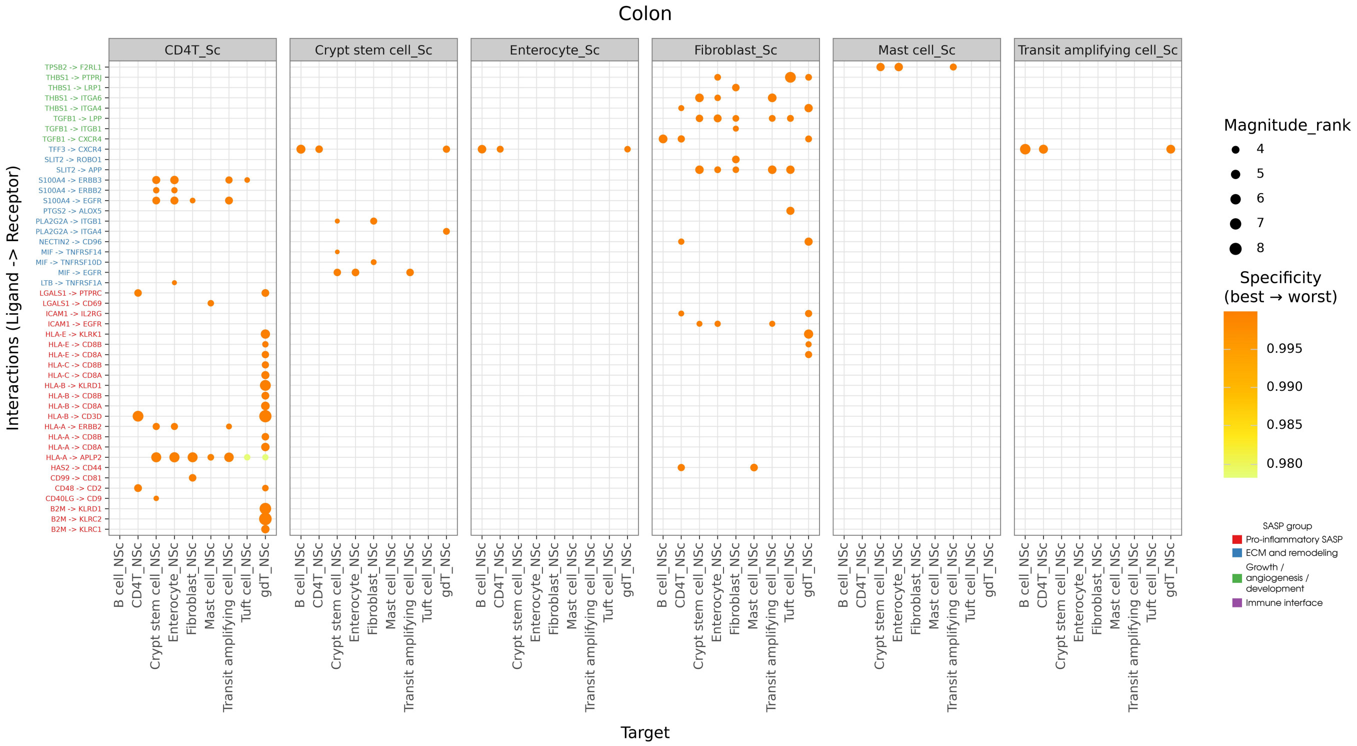

Figure S3E. Top 100 strongest interactions between Sc and NSc cells in Colon. For visualization, magnitude_rank and specificity_rank values were inverted, such that higher values indicate greater interaction strength and specificity, respectively.

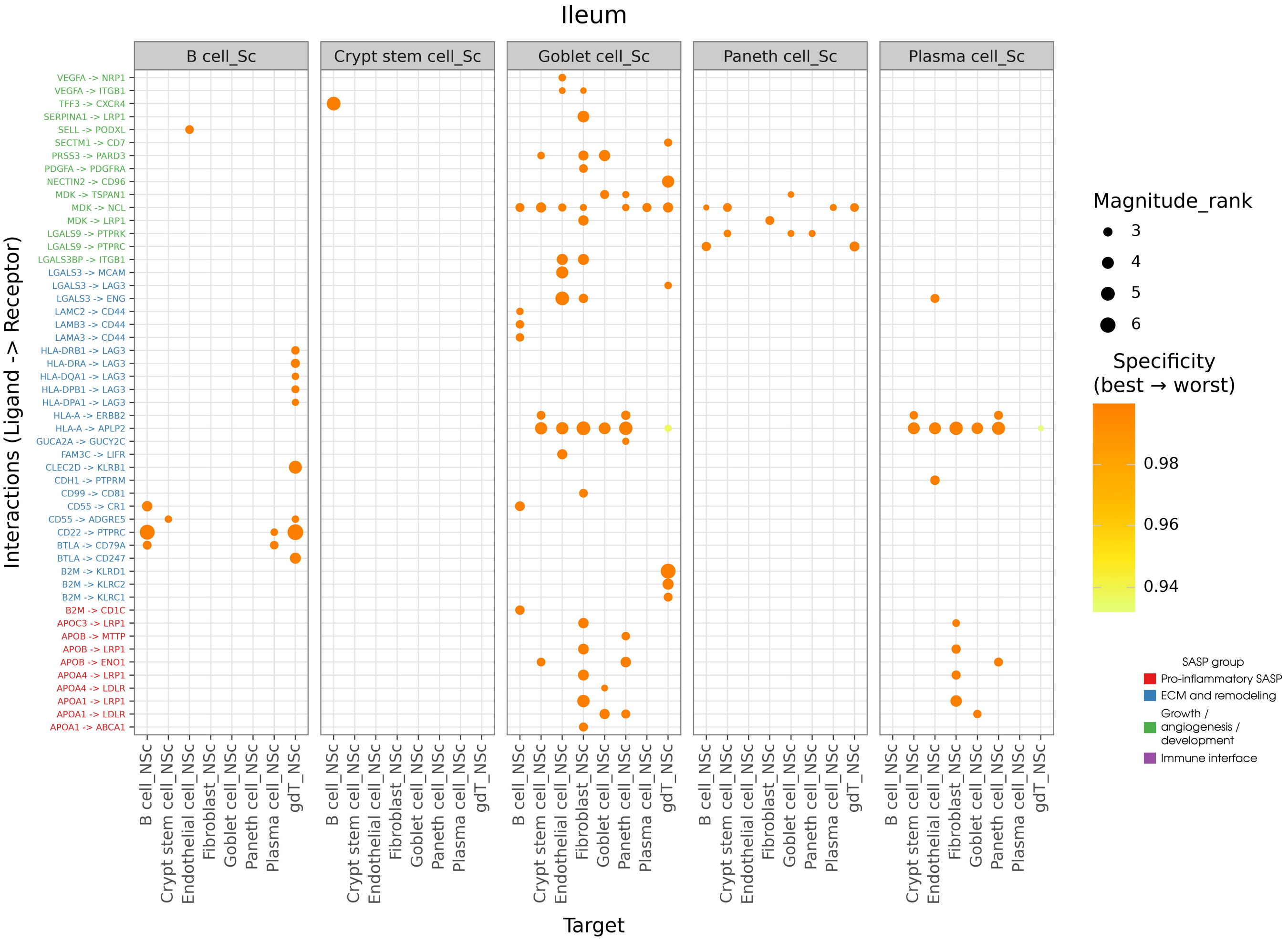

Figure S3F. Top 100 strongest interactions between Sc and NSc cells in Ileum. For visualization, magnitude_rank and specificity_rank values were inverted, such that higher values indicate greater interaction strength and specificity, respectively.

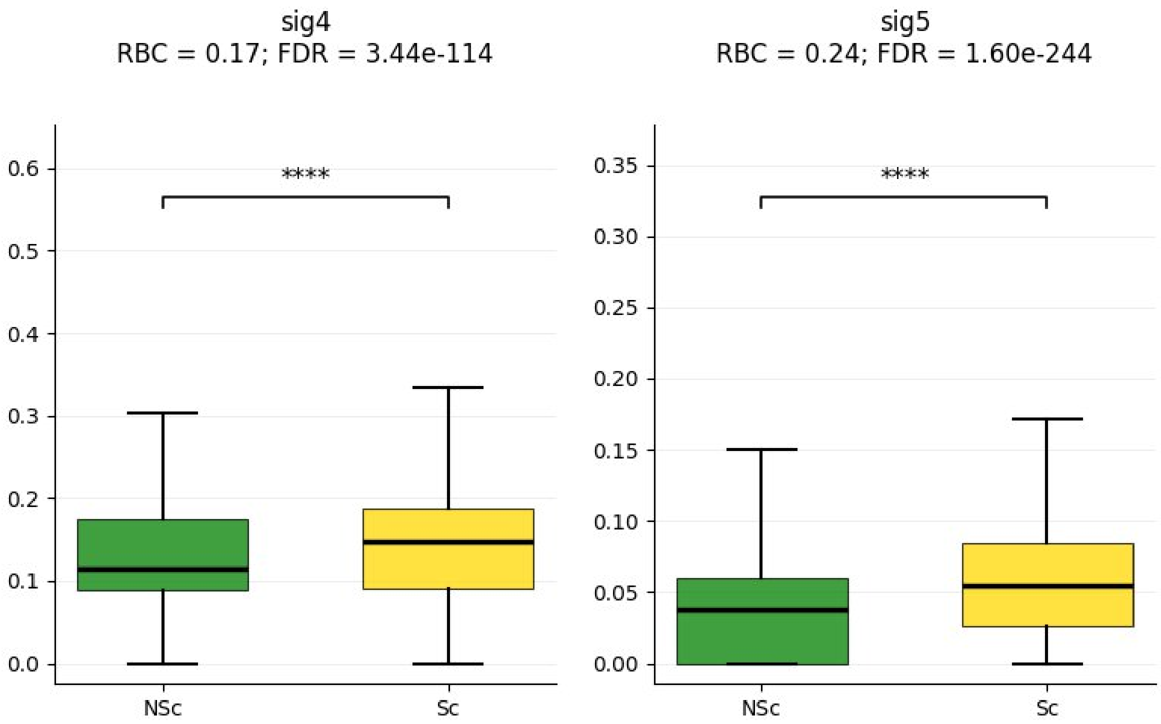

Figure S4A. AUCell scores computed at single-cell resolution for cardiomyocyte-specific gene signatures in non-senescent (NSc) and senescent (Sc) cells. Sig4 - cardiomyocyte metabolic and oxidative stress response; sig5 - senescence-associated stress response. Boxes represent the interquartile range (IQR; 25th–75th percentiles), with the horizontal line indicating the median. Whiskers extend to the most extreme values within 1.5 × IQR of the lower and upper quartiles. Statistical differences between NSc and Sc cells were assessed using a two-sided Mann–Whitney U test. Effect size was quantified using the rank-biserial correlation coefficient (RBC). P-values were adjusted for multiple testing using the Benjamini–Hochberg method.

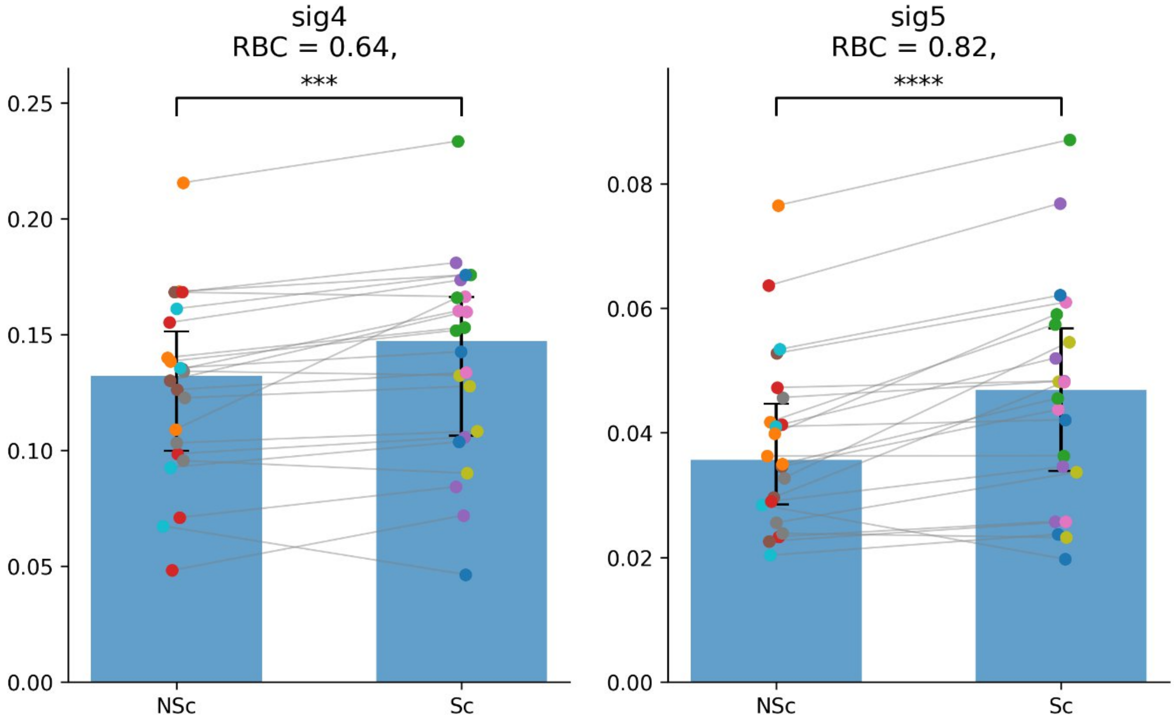

Figure S4B. AUCell scores aggregated at the donor level for cardiomyocyte-specific gene signatures in non-senescent (NSc) and senescent (Sc) cells. Sig4 - cardiomyocyte metabolic and oxidative stress response; sig5 - senescence-associated stress response. AUCell scores were averaged across cells for each donor and senescence state. Data are shown as median AUCell scores, with whiskers indicating the interquartile range (IQR; 25th–75th percentiles). Individual points represent donor-level values, and lines connect paired NSc and Sc measurements from the same donor. Statistical differences between paired NSc and Sc measurements were assessed using the two-sided Wilcoxon signed-rank test. Effect size was quantified using the paired rank-biserial correlation coefficient (RBC). P-values were adjusted for multiple testing using the Benjamini–Hochberg method.
